# Evidence for Persistence of Ectromelia Virus in Inbred Mice, Recrudescence Following Immunosuppression and Transmission to Naïve Mice

**DOI:** 10.1371/journal.ppat.1005342

**Published:** 2015-12-23

**Authors:** Isaac G. Sakala, Geeta Chaudhri, Anthony A. Scalzo, Preethi Eldi, Timothy P. Newsome, Robert M. Buller, Gunasegaran Karupiah

**Affiliations:** 1 Department of Immunology and Infectious Disease, John Curtin School of Medical Research, Australian National University, Canberra, Australia; 2 Immunology and Virology Program, Centre for Ophthalmology & Visual Science, The Lions Eye Institute, University of Western Australia, Perth, Western Australia, Australia; 3 School of Molecular Bioscience, University of Sydney, Sydney, Australia; 4 Saint Louis University Health Sciences Center, Department of Molecular Microbiology and Immunology, St Louis, Missouri, United States of America; University of Alberta, CANADA

## Abstract

Orthopoxviruses (OPV), including variola, vaccinia, monkeypox, cowpox and ectromelia viruses cause acute infections in their hosts. With the exception of variola virus (VARV), the etiological agent of smallpox, other OPV have been reported to persist in a variety of animal species following natural or experimental infection. Despite the implications and significance for the ecology and epidemiology of diseases these viruses cause, those reports have never been thoroughly investigated. We used the mouse pathogen ectromelia virus (ECTV), the agent of mousepox and a close relative of VARV to investigate virus persistence in inbred mice. We provide evidence that ECTV causes a persistent infection in some susceptible strains of mice in which low levels of virus genomes were detected in various tissues late in infection. The bone marrow (BM) and blood appeared to be key sites of persistence. Contemporaneous with virus persistence, antiviral CD8 T cell responses were demonstrable over the entire 25-week study period, with a change in the immunodominance hierarchy evident during the first 3 weeks. Some virus-encoded host response modifiers were found to modulate virus persistence whereas host genes encoded by the NKC and MHC class I reduced the potential for persistence. When susceptible strains of mice that had apparently recovered from infection were subjected to sustained immunosuppression with cyclophosphamide (CTX), animals succumbed to mousepox with high titers of infectious virus in various organs. CTX treated index mice transmitted virus to, and caused disease in, co-housed naïve mice. The most surprising but significant finding was that immunosuppression of disease-resistant C57BL/6 mice several weeks after recovery from primary infection generated high titers of virus in multiple tissues. Resistant mice showed no evidence of a persistent infection. This is the strongest evidence that ECTV can persist in inbred mice, regardless of their resistance status.

## Author Summary

Orthopoxviruses (OPV) cause acute infections in mammalian hosts but some OPV, including ectromelia virus (ECTV), have been isolated from tissues of several species of animals long after infection. We present evidence that ECTV causes a persistent infection in some strains of disease-susceptible mice in which infectious virus was present in the bone marrow for several weeks post-infection. While an antiviral response was generated and persisted during the entire study period, it was insufficient to eliminate virus. Both host factors and virus-encoded host response modifiers influenced virus persistence. Se veral weeks after infection, mice that had apparently recovered succumbed to disease and transmitted virus to co-housed naïve animals following immunosuppression. Significantly, infectious virus was also isolated from resistant mice that had been subjected to sustained immunosuppression several weeks post-infection. This is the strongest evidence that ECTV can persist in inbred mice, regardless of their resistance status.

## Introduction

An acute viral infection can result in complete recovery of the host, death or establishment of persistence. The OPV genus is generally believed to cause acute infections. However, some members such as ECTV [1–7], monkeypox virus (MPXV) [8], cowpox virus (CPXV) [8–10] and vaccinia virus (VACV) [11,12] have been reported to persist for several weeks or months after experimental infection in a variety of animal species that show no clinical signs of disease [13]. Those reports have neither been thoroughly investigated nor their significance understood. If proven correct, they have profound implications for the ecology of OPV and the epidemiology of diseases they cause. One suggestion is that the reports may be a reflection of persistent infection within a population rather than virus persistence in individual animals [13]. VARV causes smallpox in humans but the disease was successfully eradicated through vaccination more than 35 years ago [13] without any evidence of re-emergence, implying that it does not cause persistent infections.

Despite the eradication of smallpox, there is still significant interest in the pathogenesis of OPV infections due to the potential threat of accidental or intentional release of VARV [14], the emergence of zoonotic MPXV [15–17], outbreaks of VACV infection in dairy cattle and their transmission to humans [18,19] and sporadic outbreaks of cowpox in humans and various animal species [20–22]. While outbreaks of CPXV or VACV infections in humans are not common, monkeypox is an emerging disease in West and Central Africa [17,23]. The introduction of MPXV into the United States in 2003 in a consignment of wild-caught animals from Africa established for the first time that outbreaks of human monkeypox could occur outside of the African continent [24].

Mousepox is a disease that is similar to smallpox and an excellent small animal model to study the human disease. Like the outbred human population, which exhibited varying degrees of susceptibility to smallpox [13], inbred strains of mice are either resistant or susceptible to mousepox. C57BL/6, C57BL/10, AKR and some sub-lines of 129 mice are resistant whereas A/J, DBA2, CBA/H and BALB/c mice are susceptible [4,5,25–27]. At least 4 genetic loci in the mouse genome are known to confer resistance to mousepox [27], and are associated with the generation of robust innate and adaptive immunity by the host [28–37]. Susceptible strains lack resistance alleles at these loci and the immune response generated against ECTV is weak and delayed [27,33]. In resistant strains, the potent immune response can largely overcome the effects of host response modifiers (HRM) that ECTV encodes to subvert, dampen or evade immunity, whereas in susceptible strains virus-encoded HRM can overwhelm the sub-optimal immune responses. Nonetheless, susceptible mice can control infection with mutant viruses lacking specific HRM [38–40]. BALB/c mice infected with a deletion mutant of ECTV that does not express viral IFN-γ binding protein (vIFN-γbp) overcome the infection through augmented IFN-γ production and cell-mediated immunity [38]. Although the animals apparently recover from infection, preliminary studies revealed that virus is not completely eliminated. This finding provided us with an opportunity to address OPV persistence using the ECTV model.

We report here that at sub-lethal doses of wild type (WT) or mutant ECTV infection in disease-susceptible BALB/c mice, low numbers of virus genomes persisted over several weeks despite the presence of effector CD8 T cell responses. Virus genomes were detected in several organs but only in the BM and blood beyond 37 days post-infection (p.i). Importantly, infectious virus was also demonstrable in the BM of some mice more than 100 days p.i. Contemporaneous with the presence of virus, a change in the immunodominance hierarchy of CD8 T cell responses was evident during the first 3 weeks. The capacity of virus to persist was influenced by the host immune response and virus-encoded HRM. However, treatment of mice that had apparently recovered from infection with the immunosuppressive drug cyclophosphamide (CTX) several weeks post-infection caused mousepox with high titers of virus in visceral organs. CTX-treated mice, but not untreated animals, transmitted virus to co-housed naïve mice, all of which succumbed to mousepox. An unexpected and surprising finding was that treatment of the resistant C57BL/6 mice several weeks after infection with CTX also caused mousepox with high titers of virus in organs. There is no evidence of ECTV causing a persistent infection in this strain. Our data provide robust evidence that ECTV can persist at very low levels in both resistant and susceptible strains of mice.

## Results

### ECTV-IFN-γbpΔ-infected BALB/c mice recover from infection despite virus persistence

**Fig 1:**
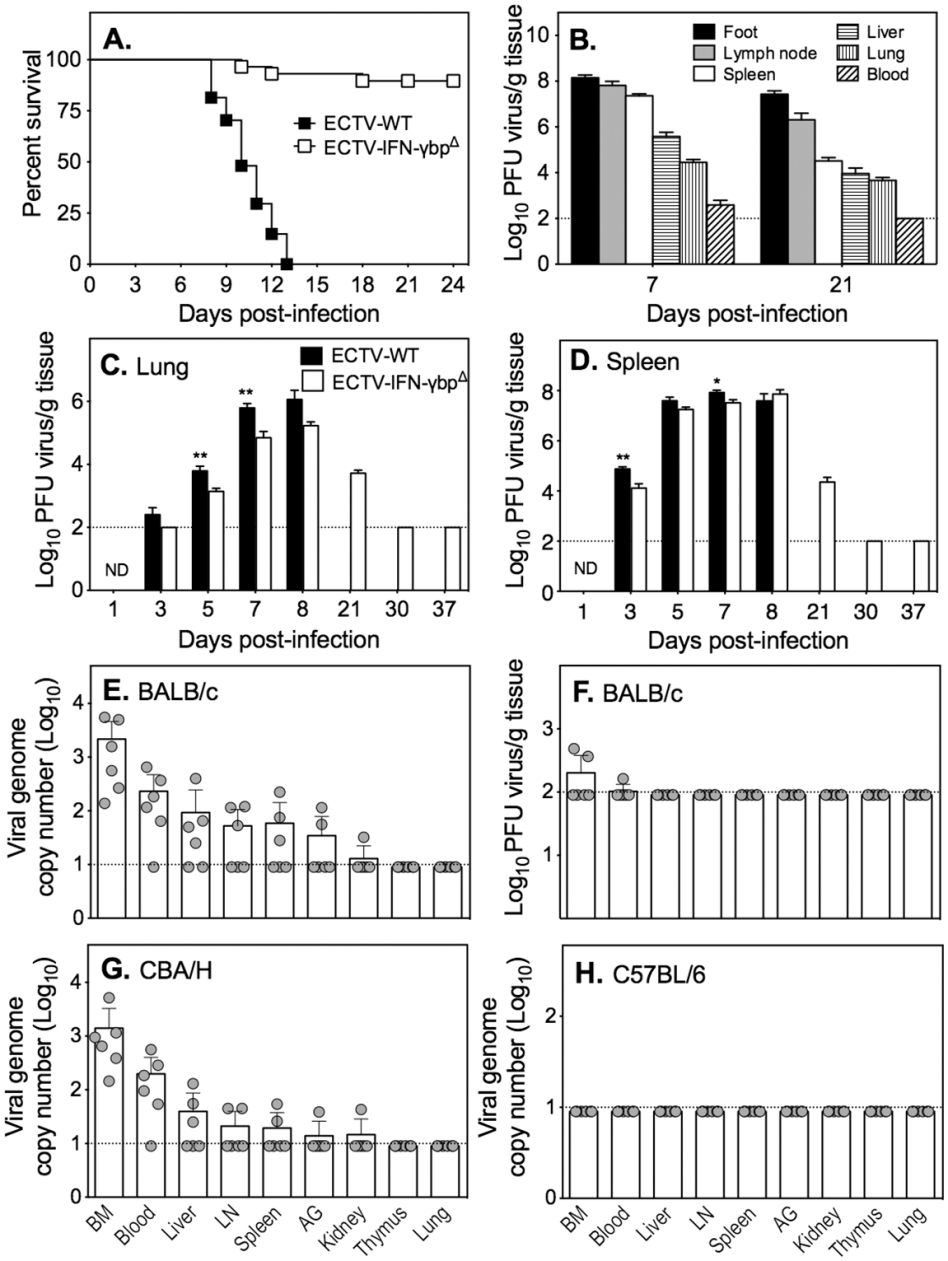
Presence of ECTV-IFN-γbp^Δ^ in mice that survive infection. (A) Survival proportions of BALB/c mice infected with 500 PFU ECTV-WT (n = 27) or ECTV-IFN-γbp^Δ^ (n = 58). *P*<0.0001, Kaplan-Meir log rank statistical test. Survival data combined from 4 separate experiments, with 6–15 mice per experiment. (B) ECTV-IFN-γbp^Δ^ titers in the indicated organs of BALB/c mice at days 7 and 21 p.i. (C) Lung and (D) spleen titers of ECTV-WT and ECTV-IFN-γbp^Δ^. *, *P*<0.05; **, *P*<0.01 in comparing ECTV-WT and ECTV-IFN-γbp^Δ^ titers at the days p.i. Data shown in (B), (C) and (D) are composite viral loads determined in individual animals from 3 different experiments with groups of 3–6 mice and expressed as means of log_10_ PFU/gram tissue ± SEM. The limit of virus detection is 2 log_10_ PFU, shown by the dotted line. (E) ECTV genomes and (F) infectious virus in organs of BALB/c mice 37 days p.i. with ECTV-IFN-γbp^Δ^. (G) ECTV genomes in organs of CBA/H mice 37 days p.i. with 500 PFU of ECTV-IFN-γbp^Δ^. (H) ECTV genomes in organs of C57BL/6 mice 37 days p.i. with 500 PFU of ECTV-IFN-γbp^Δ^. For E, F, G and H, n = 5 mice. The limit of virus genome detection is 10 copies, shown by the dotted line. BM = bone marrow; LN = popliteal lymph node; AG = adrenal gland.

BALB/c mice infected with ECTV-WT at a dose of 500 PFU or greater succumb to mousepox due to uncontrolled virus replication [33,38]. When inoculated with a similar dose of ECTV-IFN-γbp^Δ^, this strain generates good cell-mediated immunity and antibody responses with a significant proportion of animals overcoming infection but a small subset succumbing to disease ([38] and Fig 1A). Virus was isolated from most organs as late as day 21 p.i. (Fig 1B) but was below the level of detection by viral plaque assay at later time points (Fig 1C and 1D; [38]). The more sensitive qRT-PCR assay, however, revealed the presence of ECTV-IFN-γbp^Δ^ genomes in several organs at 37 days p.i., with genome copy numbers highest in the BM and blood (Fig 1E). The presence of virus genomes was biologically significant as infectious virus was detected in 2 of the 6 animals in the BM and in 1 of 6 animals in blood by viral plaque assay (Fig 1F). Virus genomes were also detected in several tissues of CBA/H mice (Fig 1G), another ECTV-susceptible strain, but not in the resistant C57BL/6 strain (Fig 1H). Despite the presence of virus genomes, the animals did not display any clinical signs of disease.

### Persistence of ECTV-specific CD8 T cell responses and inversion of immunodominance hierarchy

As animals that survived infection with ECTV-IFN-γbp^Δ^ generate neutralizing antibody responses between days 14–37 p.i. [38], we hypothesized that ineffective virus clearance might be related to suboptimal or defective CTL responses. We therefore characterized the CD8 T cell responses to ECTV-IFN-γbp^Δ^ during the early (day 7), intermediate (day 14) and late (day 21) phases of a primary infection. We used ECTV-WT and the highly attenuated ECTV-TK^Δ^, in which the viral thymidine kinase (TK) gene had been deleted, as controls for the early time point. BALB/c mice infected with ECTV-WT succumb to mousepox before day 14 whereas ECTV-TK^Δ^ is cleared rapidly and CD8 T cell responses are not detectable at day 14 or beyond.

**Fig 2:**
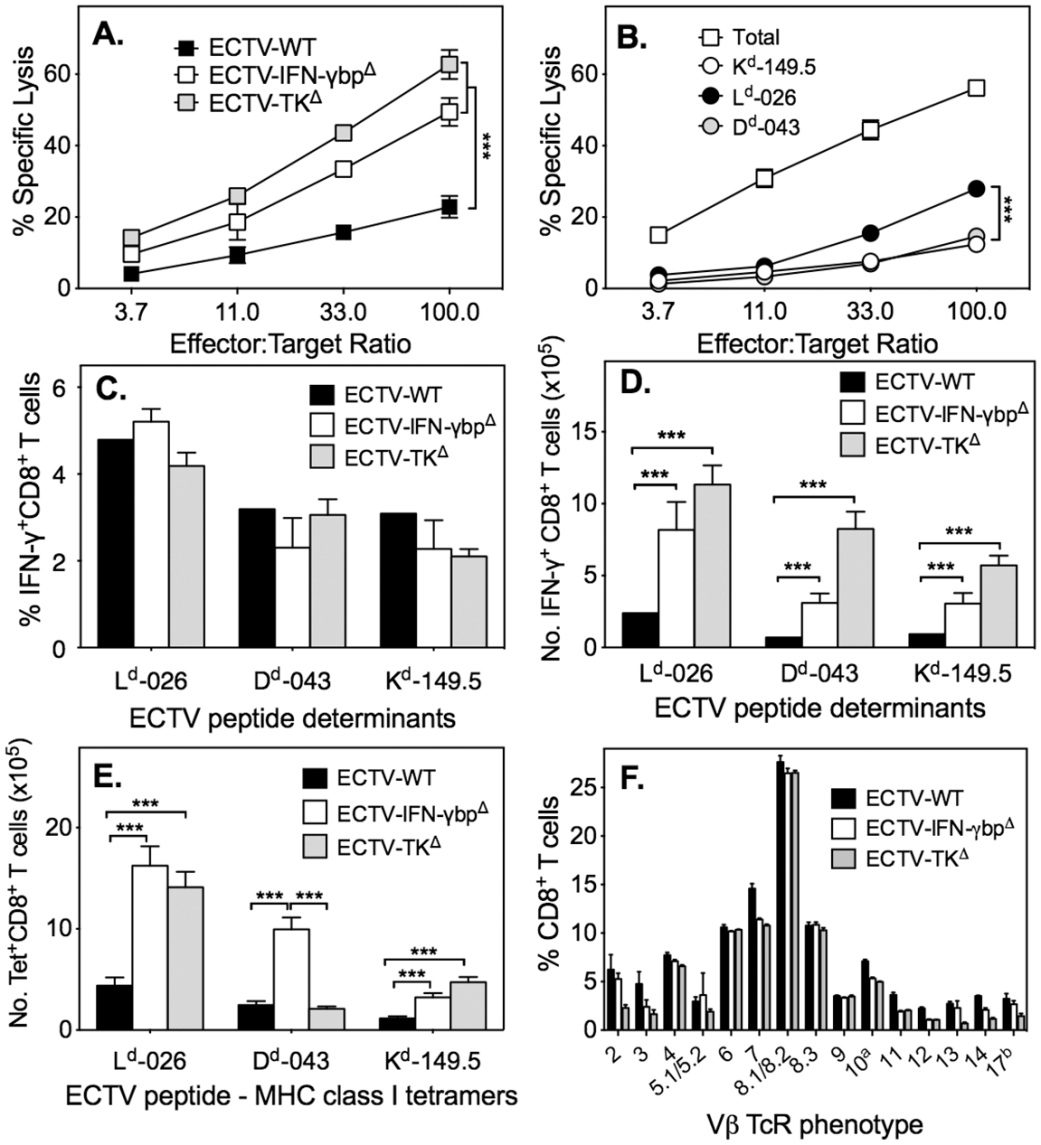
CD8 T cell responses during acute ECTV infection. Groups of female BALB/c mice were infected with 500 PFU ECTV-IFN-γbp^Δ^, 500 PFU ECTV-WT or 2 x 10^6^ PFU ECTV-TK^Δ^ s.c., sacrificed on day 7 p.i. and splenic antigen-specific CD8 T cell responses were measured. (A) Percent specific lysis of ECTV-infected, ^51^Cr-labelled P815 targets by splenocytes from infected mice. ***, *P*<0.001 in comparing % specific lysis of virus-infected target cells by splenocytes from ECTV-WT infected mice with splenocytes from ECTV-IFN-γbp^Δ^- or ECTV-TK^Δ^-infected mice at the indicated effector-to-target ratios. (B) Percent specific lysis of virus-infected (Total) or ECTV CD8 T cell determinant-pulsed, ^51^Cr-labelled P815 targets by splenocytes from ECTV-IFN-γbp^Δ^-infected mice. **, *P*<0.01 and ***, *P*<0.001 in comparing % specific lysis of L^d^-026 peptide-pulsed targets with D^d^-043 or K^d^-149.5 peptide-pulsed targets by splenocytes from ECTV-IFN-γbp^Δ^-infected mice at the indicated effector-to-target ratios. (C) Percent ECTV peptide determinant-specific IFN-γ^+^ CD8 T cells in spleens of ECTV-IFN-γbp^Δ^-infected mice. L^d^-026-restricted responses were significantly higher (*P*<0.01) compared with D^d^-043- or K^d^-149.5-restricted responses. (D) Numbers of ECTV-specific IFN-γ^+^ CD8 T cells in spleens of ECTV-IFN-γbp^Δ^-infected mice. ***, *P*<0.001 for comparisons between groups shown. (E) Numbers of peptide-MHC class I tetramer^+^ CD8 T cells in spleens of ECTV-IFN-γbp^Δ^-infected mice. ***, *P*<0.001 for comparisons between groups shown. (F) TCR Vβ chain expression by splenic CD8 T cells from virus-infected mice. *P* values for all panels were obtained by Mann-Whitney U test.

At day 7 p.i., ECTV-TK^Δ^ and ECTV-IFN-γbp^Δ^ induced CTL responses that were comparable but about 9-fold higher in magnitude than the response elicited by ECTV-WT (Fig 2A) [38]. The determinant-specific CTL response induced by ECTV-IFN-γbp^Δ^ (Fig 2B) was also similar to that generated by ECTV-TK^Δ^ but the response was 3-9-fold lower in ECTV-WT-infected mice (S1 Fig). The L^d^-026 determinant-specific response was the strongest followed by D^d^-043- and K^d^-149.5-specific responses.

Each of the viruses induced similar proportions of determinant-specific IFN-γ^+^ CD8 T cells, with a greater proportion of cells responding to L^d^-026 peptide (Fig 2C). However, in terms of numbers, ECTV-TK^Δ^ and ECTV-IFN-γbp^Δ^ induced significantly higher determinant-specific IFN-γ^+^ CD8 T cells compared to ECTV-WT (Fig 2D). The L^d^-026-specific tetramer^+^ CD8 T cells were the predominant population (Fig 2E) followed by the D^d^-043 and K^d^-149.5 tetramer^+^ CD8 T cells. Consistent with the determinant-specific IFN-γ response, the numbers of tetramer^+^ CD8 T cells induced by ECTV-WT were significantly fewer compared to numbers in mice infected with the other viruses. All 3 viruses also induced similar T cell receptor (TCR) Vβ chain repertoires in CD8 T cells at day 7 p.i., with greater than 35% expressing the TCR Vβ8, followed by Vβ7, Vβ6, Vβ4 and Vβ10a (Fig 2F). Thus, although all 3 viruses induced CD8 T cells of comparable TCR Vβ repertoires, ECTV-WT induced a poor response in terms of numbers and functionality.

**Fig 3:**
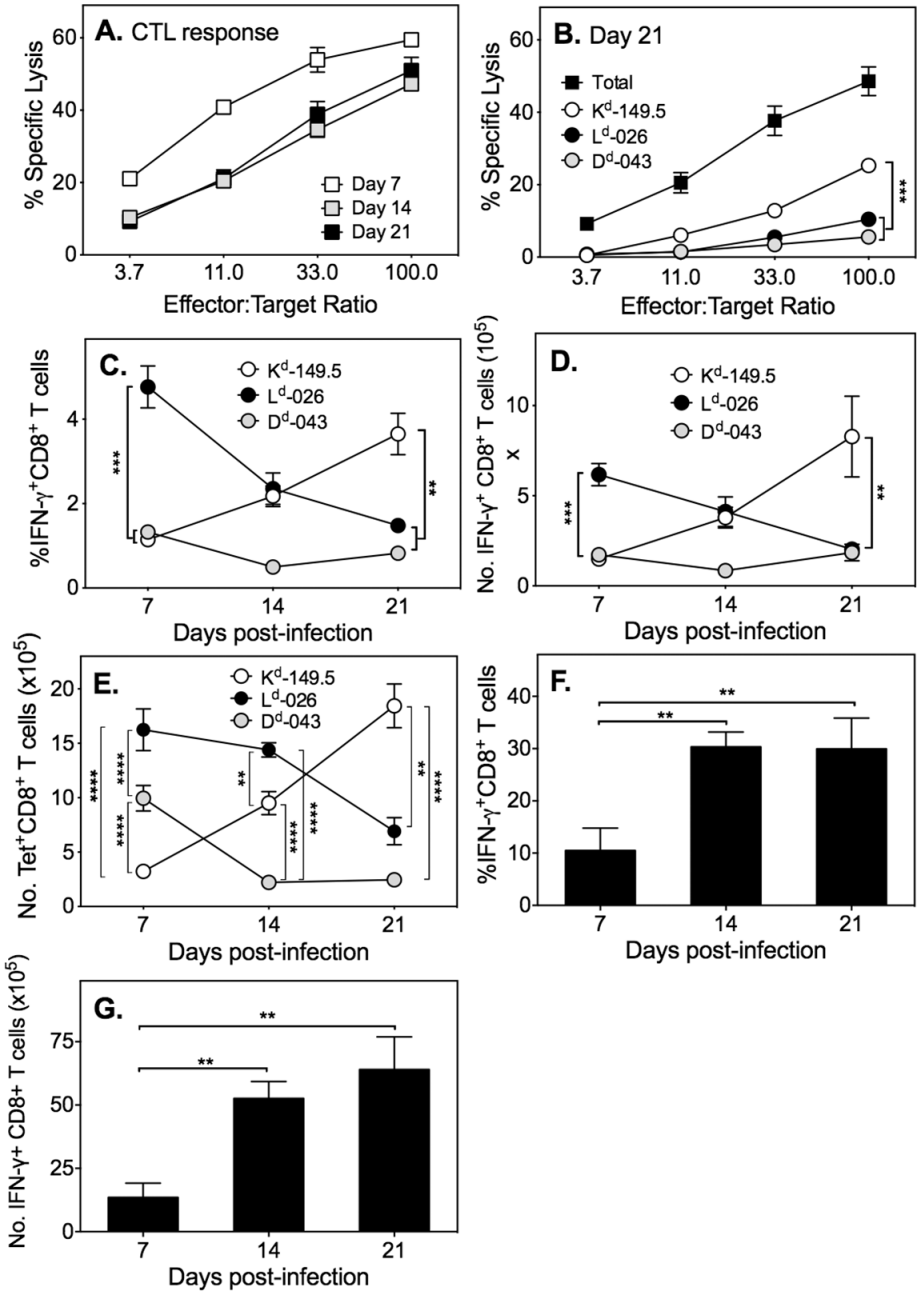
Antigen-specific CD8 T-cell responses at days 14 and 21 p.i. Groups of 5 female BALB/c mice were infected with 500 PFU of ECTV-IFN-γbp^Δ^ and their spleen cells used to measure CD8 T cell responses. (A) *Ex vivo* cytolytic activity of splenocytes from ECTV-IFN-γbp^Δ^ infected mice against virus-infected P815 target cells on days indicated p.i. (B) *Ex vivo* cytolytic activity of splenocytes obtained from ECTV-IFN-γbp^Δ^ infected mice 21 days p.i. against ECTV-infected (Total) or ECTV peptide determinant-pulsed P815 target cells. ***, *P*<0.001. (C) Percent of ECTV peptide determinant-specific IFN-γ^+^ CD8 T cells. **, *P*<0.01 and ***, *P*<0.001. (D) Numbers of ECTV peptide determinant-specific IFN-γ^+^ CD8 T cells. **, *P*<0.01 and ***, *P*<0.001. (E) Numbers of peptide-MHC class I tetramer^+^ CD8 T cells. **, *P*<0.01 and ****, *P*<0.0001. (F) Percent ECTV-specific (total) IFN-γ^+^ CD8 T cells. (G) Numbers of ECTV-specific (total) IFN-γ^+^ CD8 T cells. **, *P*<0.01. *P* values for all panels were obtained by Mann-Whitney U test.

At 2 and 3 weeks p.i., virus-specific cytotoxic T lymphocyte (CTL) activity was demonstrable in mice infected with ECTV-IFN-γbp^Δ^, albeit the magnitude was about 3-fold lower than day 7 (Fig 3A). By day 21 p.i., the response was predominantly K^d^-149.5-restricted (Fig 3B), associated with increased proportions (Fig 3C) and numbers (Fig 3D) of K^d^-149.5-specific IFN-γ^+^ cells and K^d^-149.5 tetramer^+^ (Fig 3E) CD8 T cells. Proportions (Fig 3F) and numbers (Fig 3G) of ECTV-specific (total) IFN-γ^+^ CD8 T cells increased at day 14 p.i., over and above day 7 p.i., and remained high at day 21. The dominance of K^d^-149.5 tetramer^+^ CD8 T cells (S2A Fig) and K^d^-149.5-restricted CTL activity at day 21 (S2B Fig) also occurs in mice infected with a sub-lethal dose (100 PFU) of ECTV-WT and is therefore not unique to ECTV-IFN-γbp^Δ^ infection. At this dose, ECTV-WT induces similar numbers of tetramer^+^ CD8 T cells as mice infected with 500 PFU of ECTV-IFN-γbp^Δ^ (S2C Fig).

The L^d^-026 tetramer^+^ CD8 T cells were predominantly Vβ8.1/8.2^+^ at day 7 p.i., and despite a decline in proportions at days 14 and 21 p.i., they were still the dominant type (S3 Fig). Conversely, proportions of K^d^-149.5 tetramer^+^ Vβ8.1/8.2^+^ CD8 T lymphocytes expanded gradually from day 7 and were the main type by day 21 p.i., with proportions of Vβ10^a+^ and Vβ11^+^ cells also increasing by this time. The D^d^-043 tetramer^+^ CD8 T cells utilized the Vβ8.1/8.2 TCR repertoire early in infection but to a lesser extent at later time points. While the significance of TCR Vβ chain usage by CD8 T cells in BALB/c mice or with respect to ECTV persistence is currently unknown, it is notable that the most responsive CD8 T cell populations utilized the Vβ8.1/8.2 TCR chain.

Taken together, these data indicate the presence of activated, effector CD8 T cells and an inversion of immunodominance hierarchy of the CD8 T cell response during the first 3 weeks p.i. An investigation into the mechanism(s) that result in a change in the immunodominance hierarchy of the CD8 T cell response is beyond the scope of this study but the results are consistent with virus persistence.

### Activation and persistence of virus-specific CD8 T cells correlates with presence of virus genomes

CD62L, the homing receptor for lymphocytes and CD127, the IL-7 receptor α chain are both expressed at high levels on the surface of naïve CD8 T cells but are reduced following antigenic stimulation and activation. Low-level virus persistence (Fig 1E and 1F) might have been responsible for an ongoing immune response as demonstrated by reduced expression of CD62L and CD127 (S4A Fig), IFN-γ production (S4B Fig) and cytolytic activity exhibited by CD8 T cells at day 37 p.i. (S4C Fig). Significant numbers of tetramer^+^ CD8 T cells were also present at this late stage of infection (S4D Fig).

**Fig 4:**
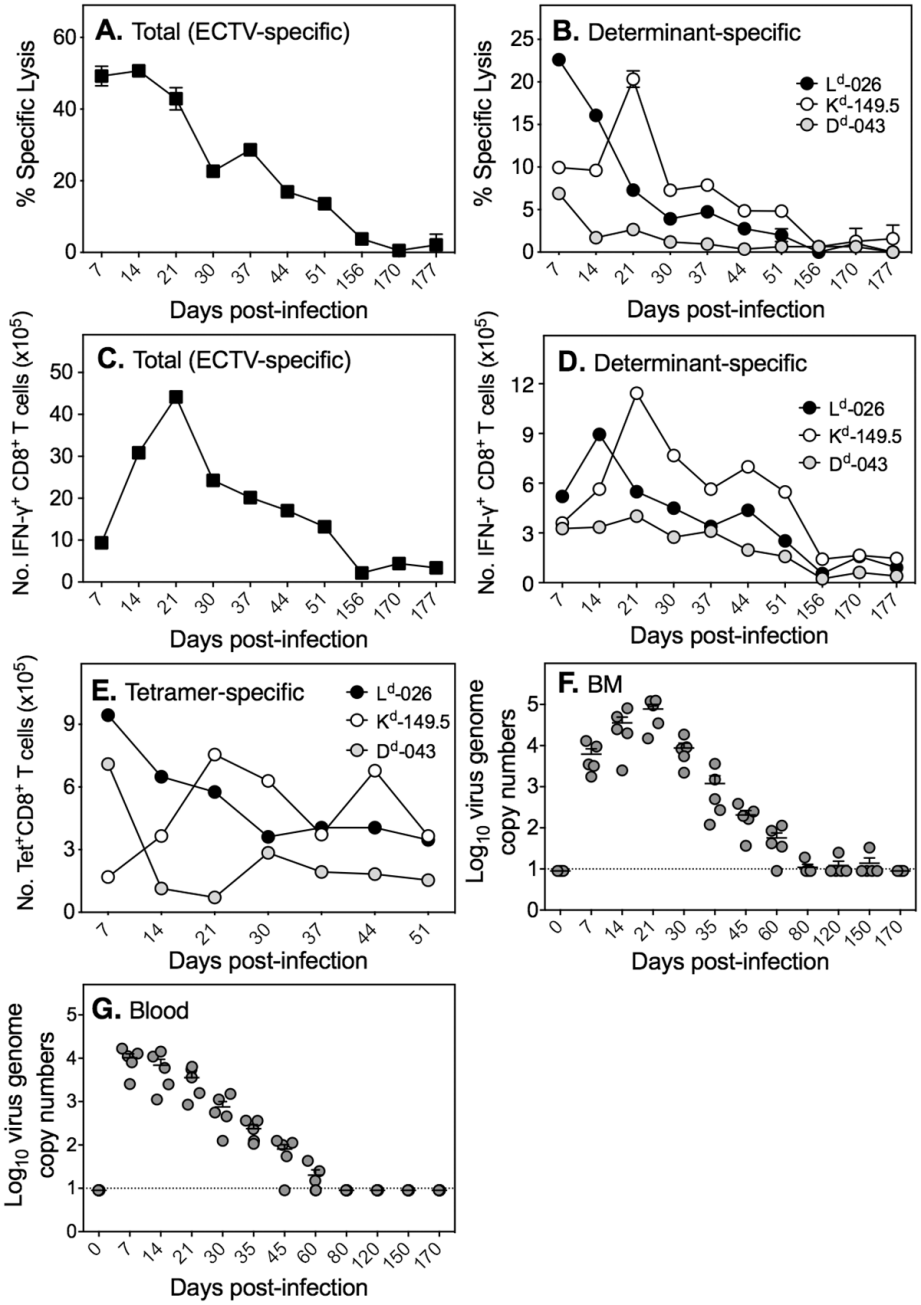
Long-term persistence of ECTV-specific CD8 T cell responses correlates with persistence of virus genomes in the BM and blood. For data shown in panels A-E, groups of 6 BALB/c mice were infected with 500 PFU ECTV-IFN-γbp^Δ^ and functional and phenotypic assays done on days 7 till day 177. (A) Percent specific lysis of ECTV-infected, ^51^Cr-labelled P815 targets by splenocytes from infected mice at 100:1 effector: target ratio. (B) Percent specific lysis of ECTV peptide determinant-pulsed, ^51^Cr-labelled P815 targets by splenocytes from infected mice at 100:1 effector: target ratio. (C) Kinetics of ECTV-specific (total) IFN-γ^+^ CD8 T cells numbers. (D) Kinetics of ECTV peptide determinant-specific IFN-γ^+^ CD8 T cell numbers. (E) Kinetics of peptide-MHC class I tetramer^+^ CD8 T cell numbers. (F) Kinetics of ECTV genome copy numbers in BM. (G) Kinetics of ECTV genome copy numbers in blood. The limit of virus genome detection is 10 copies, shown by the dotted line. Data shown in panels F and G are from the same experiment in which 5 mice infected with 500 PFU ECTV-IFN-γbp^Δ^ were sacrificed on the indicated days but qRT-PCR assay run on the same day.

In ECTV-resistant C57BL/6 mice, the kinetics of viral load in organs closely parallels the CTL activity over the first 2 weeks but the response contracts and is undetectable once virus is cleared [33,34]. However, in C57BL/6 mice lacking B or CD4 T lymphocytes, ECTV causes a persistent infection and chronic stimulation of CTL activity [34]. Thus, the presence of virus (or viral antigen) appears necessary for continued stimulation of CD8 T cells. We reasoned that the presence of activated effector CD8 T cells in BALB/c mice beyond day 37 p.i. might be indicative of viral antigenic stimulation of this population and possibly virus persistence. Indeed, virus- (Fig 4A) and determinant-specific (Fig 4B) CTL responses were detectable throughout the 177-day period in ECTV-IFN-γbp^Δ^-infected mice. The responses were high during the first 3 weeks p.i., consistent with data in Figs 2 and 3, with a gradual decline over time but still detectable at very low levels at day 177. The kinetics of the CTL response corresponded with virus- (Fig 4C) and determinant-specific (Fig 4D) IFN-γ^+^ and tetramer^+^ CD8 T cell numbers (Fig 4E) and proportions (S5 Fig). This longer-term study confirmed that there was a change in the immunodominance hierarchy of the CD8 T cell response during the first 3 weeks p.i. The kinetics of the CD8 T cell responses closely paralleled the presence of virus genomes in the BM (Fig 4F) and blood (Fig 4G), at least over the first 60 days p.i. Virus genomes were largely below the limit of detection after this period, but low levels of genome copies were occasionally detectable in the BM of a very small number of animals. Despite our inability to detect ECTV genomes consistently beyond 60 days p.i, low-level CD8 T cell effector activity was still measurable (Fig 4A–4E), suggesting that viral antigen was likely being presented to the T cells. However, these effector cells were unable to completely clear the persistent virus infection.

### Virus and host factors influence virus persistence

Numerous mechanisms can potentially contribute to virus persistence but at least two important factors are the effectiveness of host immune response in eliminating virus and subversion of the response by virus-encoded HRM. We investigated the roles of 4 specific virus-encoded HRM known to dampen cell-mediated immunity in contributing to virus persistence. In addition, we used BALB congenic mice to assess whether some host genes associated with genetic resistance to mousepox and known to modulate cell-mediated immunity contribute to overcoming virus persistence.

Deletion of vIFN-γbp reduced viral load and increased survival rates of BALB/c mice, but virus genomes were still detectable at day 37 p.i. (Fig 1). We reasoned that deletion of additional genes encoding HRM might be more effective in clearing virus and overcoming persistence. Single deletion ECTV mutants lacking vIFN-α/βbp (ECTV-IFN-α/βbp^Δ^), vIL-18bp (ECTV-IL-18bp^Δ^) or viral serine protease inhibitor-2 (vSPI-2, an inhibitor of caspase activity) (ECTV-SPI-2^Δ^), a double mutant lacking vIFN-γbp and IL-18bp ECTV-IFN-γbp^Δ^-IL-18bp^Δ^), and a triple mutant lacking vIFN-γbp, vIL-18bp and vSPI-2 (ECTV-IFN-γbp^Δ^-IL-18bp^Δ^-SPI-2^Δ^) were used to infect BALB/c mice. Each of these HRM has been shown to dampen the host NK cell and CTL responses [38–41].

**Fig 5:**
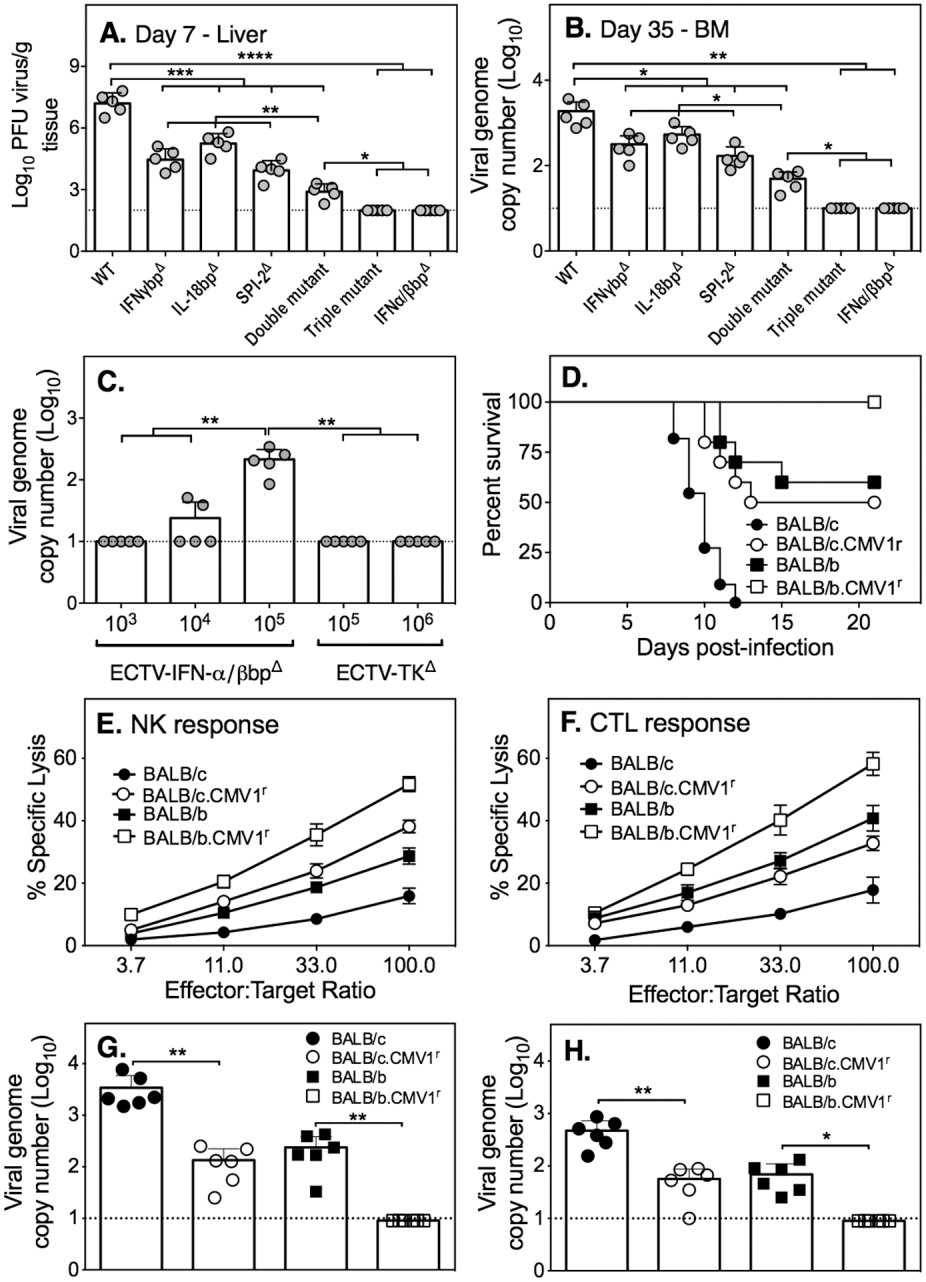
The effect of virus-encoded HRM and host resistance loci on virus persistence. Groups of 5 female BALB/c mice were infected with 100 PFU of WT, IFN-γbp^Δ^, IFN-α/βbp^Δ^, IL-18bp^Δ^, SPI-2^Δ^, IFN-γbp^Δ^-IL-18bp^Δ^ (double mutant) IFN-γbp^Δ^-IL-18bp^Δ^-SPI-2^Δ^ (triple mutant) ECTV. Separate groups of mice were sacrificed on days 7 and 35 to measure viral load. (A) Viral load in the liver at day 7 p.i. (B) Virus genome copy numbers in the BM at day 35 p.i. (C) Viral genome copy numbers in the BM of BALB/c mice 35 days p.i. with varying doses of ECTV-IFN-α/βbp^Δ^ or of ECTV-TK^Δ^. For (A), (B) and (C), *, *P*<0.05; **, *P*<0.01; ***, P<0.001; ****, *P*<0.0001. (D) Survival of WT (BALB/c, BALB/b) and congenic (BALB/c.*Cmv*1^r^, BALB/b.*Cmv*1^r^) mice infected with 500 PFU ECTV-WT. *P* values were obtained by Log-rank (Mantel-Cox) test: *P*<0.001 in comparing % survival of BALB/c with BALB/c.*Cmv*1^r^; *P*<0.05 in comparing % survival of BALB/b with BALB/b.*Cmv*1^r^; *P*<0.0001 in comparing % survival of BALB/c with BALB/b; *P*<0.05 in comparing % survival of BALB/c.*Cmv*1^r^ with BALB/b. (E) NK cell and (F) CTL responses at day 7 p.i. in WT and congenic mice infected with 100 PFU ECTV-WT. (G) Virus genome copy numbers in the BM at day 35 p.i. in WT and congenic mice infected with 100 PFU ECTV-WT. (H) Virus genome copy numbers in the BM at day 35 p.i. in WT and congenic mice infected with 10^5^ PFU of ECTV-IFN-α/βbp^Δ^. The limit of virus detection in A is 2 log_10_ PFU, shown by a dotted line. The limit of virus genome detection in B, C, G and H is 10 copies, shown by the dotted line. For panels A-C, G and H, *P* values were obtained by Mann-Whitney U test for the indicated comparisons: *, *P*<0.05, **, *P*<0.01, ***, *P*<0.001 and ****, *P*<0.0001.

Titers of single mutant viruses were significantly lower in the liver at day 7 (Fig 5A) compared with ECTV-WT and viral load was further reduced when both vIFN-γbp and vIL-18bp were deleted (double mutant). The biggest reductions in viral load were evident in mice infected with the triple mutant or ECTV-IFN-α/βbp^Δ^, which were below the limit of detection. Similarly, genomes of the triple mutant and ECTV-IFN-α/βbp^Δ^ were also below the limit of detection in the BM (Fig 5B) and blood (S6A Fig) at day 35 p.i. However, a caveat to this finding is that ECTV-IFN-α/βbp^Δ^ was detectable in the liver at day 7 (S6B Fig) and in the BM at day 35 p.i (Fig 5C) when the inoculation dose was increased by 100- or 1000-fold. At similar or higher doses of the highly attenuated ECTV-TK^Δ^, virus genomes were not detectable in the BM (Fig 5C) or liver (S6B Fig). The data suggested that effective control of virus replication in the liver by day 7 significantly reduced the possibility of virus genome detection in the BM at day 35 p.i.

Of the 4 genetic loci in the mouse genome known to confer resistance to mousepox [27], resistance to mousepox 1 (*Rmp-1*) locus on chromosome 6 maps to the natural killer cell complex (NKC) [42] and *Rmp-3* locus on chromosome 17 is linked to the major histocompatibility complex (MHC) [43], and believed to be the classical MHC class Ia D^b^ molecule [26]. The ECTV-resistant C57BL/6 strain encodes all known resistance loci. In addition, the non-classical MHC class Ib molecule Qa-1^b^, bound with ECTV-derived or ECTV-induced host protein-derived peptides, can activate NK cells via the CD94-NKG2E heterodimer receptor and contribute to resistance against mousepox [44].

We speculated that BALB congenic mice encoding the C57BL/6 NKC (BALB/c.*Cmv1* ^r^) [45], MHC (BALB/b) or both (BALB/b.*Cmv*1^r^) loci would control ECTV replication more effectively and potentially overcome virus persistence. Indeed, at a dose of 500 PFU ECTV-WT, 100% of BALB/b.*Cmv1* ^*r*^ mice survived infection compared with survival rates of 60% in BALB/b strain, 50% in BALB/c.*Cmv*1^r^ mice and 0% in BALB/c mice (Fig 5D). The combined expression of *Rmp-1* and *Rmp-3* in BALB/b.*Cmv1* ^*r*^ conferred this strain a higher degree of resistance to ECTV infection compared with the other strains (Fig 5D). Nonetheless, the 4 strains of mice fully recovered from a sub-lethal dose of 100 PFU ECTV. At this dose, BALB/c.*Cm*v1^r^ and BALB/b.*Cm*v1^r^ mice generated stronger NK cell responses (Fig 5E) whereas BALB/c.*Cm*v1^r^, BALB/b and BALB/b.*Cmv1* ^*r*^ generated antiviral CTL responses (Fig 5F) that were higher in magnitude than BALB/c mice at day 7 p.i. The increased resistance of BALB/c.*Cm*v1^r^ and BALB/b.*Cm*v1^r^ congenic mice compared to the corresponding WT strains BALB/c and BALB/b, respectively) was, at least in part, due to the function of NK cells as depletion of this subset with the anti-NK1.1 mAb abolished protection (S6C Fig). In the NKC congenic strains, viral genome copy numbers were lower in the BM compared to the corresponding WT strains at day 35 (Fig 5G) and below the limit of detection in BALB/b.*Cmv*1^r^ strain.

It was of interest to determine whether infection of BALB/b.*Cmv*1^r^ congenic mice with the attenuated ECTV-IFN-α/βbp^Δ^ would further reduce the level of virus replication and tip the balance in favor of the host, allowing it to overcome virus persistence. Indeed, virus genomes were below the limit of detection in the BM of BALB/b.*Cmv*1^r^ mice at 35 days p.i. with 10^5^ PFU of ECTV-IFN-α/βbp^Δ^, whereas virus genomes were detectable in the other mouse strains (Fig 5H). Titers of ECTV-IFN-α/βbp^Δ^ were significantly lower in BALB/b and BALB/c.*Cmv*1^r^ mice compared to WT BALB/c mice. Taken together, the data suggested that both host and viral factors impact on whether virus persists.

### Immunosuppression results in active virus replication and disease

The ongoing immune response likely kept viral load under check but was insufficient to eliminate virus, suggesting equilibrium might have been reached between virus and the host immune response. We reasoned that perturbation of the equilibrium through immunosuppression might tip the balance in favor of the virus and allow the low-grade persistent infection to become an overwhelming one. In the results described below, mice that had been infected with ECTV 80 days previously were treated with CTX every 5 days over a period of 15 and monitored for a further 6–7 days. The ECTV-resistant C57BL/6 strains were treated every 5 days over a period of 20 and monitored for a further 8 days. We chose CTX treatment for immunosuppression since the combined depletion of CD4 and CD8 T cell subsets, NK cells, granulocytes and plasmacytoid dendritic cells with monoclonal antibodies [46] over 3 weeks was not sufficient to cause disease or result in virus recrudescence (S7 Fig).

**Fig 6:**
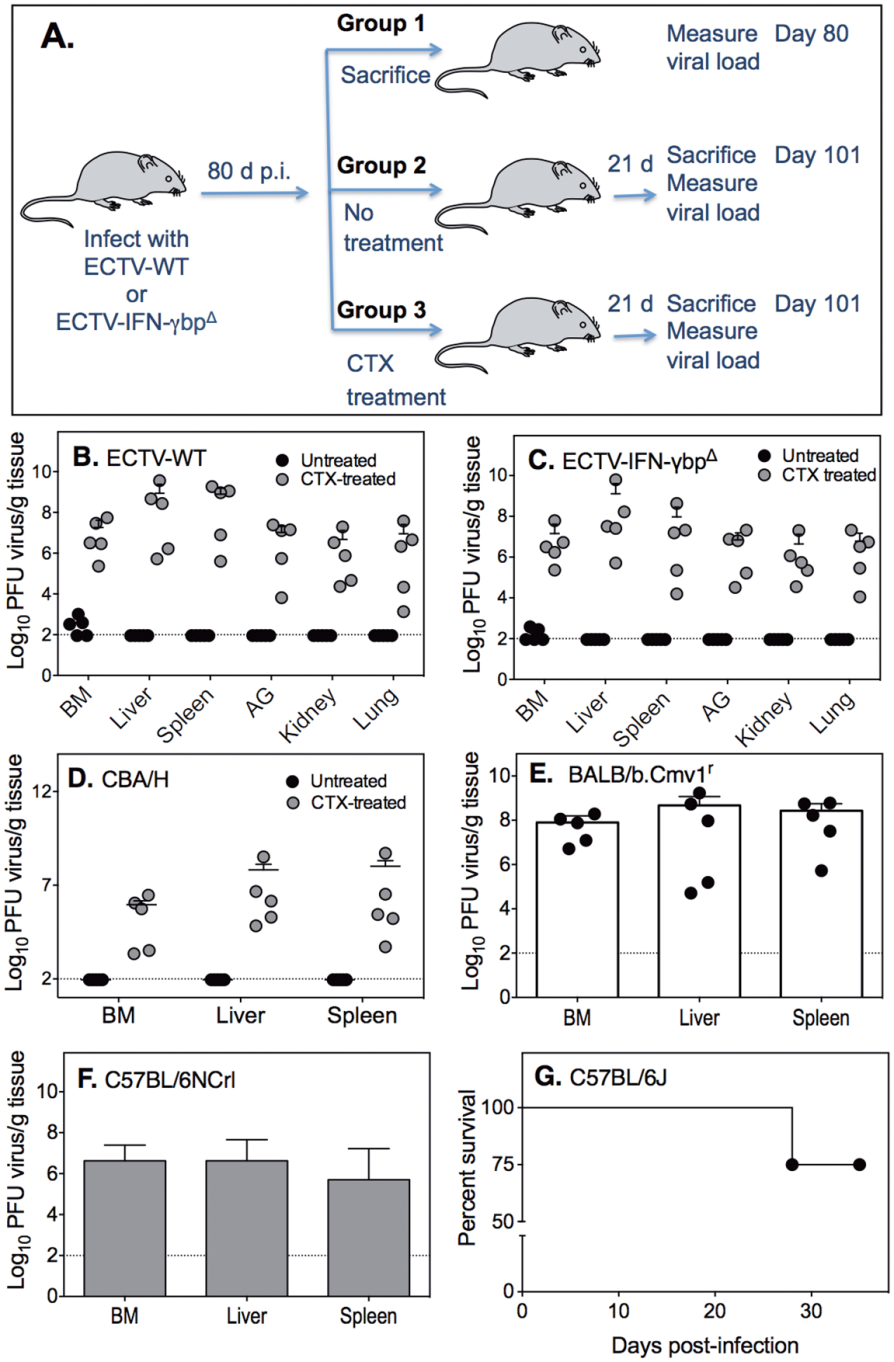
Immunosuppression with CTX triggers ECTV replication. (A) Experimental scheme. Groups of 5 WT BALB/c mice were infected with 100 PFU of ECTV-WT or ECTV-IFN-γbp^Δ^. Group 1 was sacrificed 80 days p.i. to measure viral load in various tissues, Group 2 was left untreated and Group 3 was treated with CTX. Three weeks later (day 101 p.i.), CTX-treated and untreated mice were sacrificed to measure viral load in organs. (B) ECTV-WT and (C) ECTV-IFN-γbp^Δ^ titers in organs of untreated or CTX-treated mice at 101 days p.i. BM, bone marrow; AG, adrenal gland. (D) CBA/H mice (n = 5/group) were infected with 100 PFU of ECTV-WT and 80 days p.i., one group was treated with CTX and the second group was left untreated. Shown are ECTV-WT titers in organs of untreated or CTX-treated CBA/H mice at 101 days p.i. For panels B-D, *P* <0.01 by Mann-Whitney U test for differences between untreated and CTX treated groups. (E) BALB/b.*Cmv*1^r^ mice (n = 5) were infected with 10^5^ PFU of ECTV-IFN-α/βbp^Δ^ and 80 days later treated with CTX 3 times over 15 days and sacrificed at 21 days post commencement of treatment (101 days p.i). (F) C57BL/NCrl (n = 3) and (G) C57BL/6J (n = 4) were infected with 1000 PFU of ECTV-WT and 80 days p.i., treated with CTX 4 times over 20 days. (F) ECTV-WT titers in organs of CTX-treated C57BL/6NCrl mice at 28 days post commencement of treatment (108 days p.i.) (G) Survival of C57BL/6J mice post commencement of treatment with CTX. One mouse that died on day 28 had high titers of virus in liver and spleen whereas no infectivity was detected in the organs of the remaining 3 animals that were sacrificed 35 days post commencement of treatment with CTX.

Groups of WT BALB/c mice infected with sub-lethal doses of ECTV-WT or ECTV-IFN-γbp^Δ^ were separated into 3 groups at 80 days p.i. (Fig 6A). Group 1 was sacrificed at day 80 p.i. to measure virus genomes in various tissues. Group 2 was left untreated but maintained for a further 3 weeks as a control for Group 3, which was treated with CTX every 5 days over a 3-week period. When some CTX-treated mice showed clear signs of disease or succumbed to mousepox 3 weeks later, these and control untreated animals (Group 2) were sacrificed to measure viral load in organs.

At 80 days p.i., virus genomes were not detected in the blood or visceral organs of mice in Group 1 except in the BM of 1 of 5 mice infected with ECTV-WT (S8A Fig) and 2 of 5 mice infected with ECTV-IFN-γbp^Δ^ (S8B Fig). At 101 days p.i., infectious virus was isolated from the BM of some animals but not in any other organ of ECTV-WT (Fig 6B) or ECTV-IFN-γbp^Δ^ infected mice that were not treated with CTX (Group 2) (Fig 6C). In contrast, CTX treatment of mice in Group 3 resulted in significant increases in viral load in all organs, regardless of the type of virus used for infection (Fig 6B and 6C). In a similar but separate experiment, CTX treatment over 3 weeks also resulted in high ECTV titers in organs of CBA/H mice 85 days p.i. with ECTV-WT (Fig 6D), indicating that virus persistence is not unique to the BALB/c strain.

It is of note that virus genomes were not detected in the BM of most animals sacrificed at day 80 (S8A and S8B Fig), but infectious virus was isolated from all similarly infected animals that were treated with CTX for 3 weeks (Fig 6B and 6C). Thus, failure to detect virus genomes in the BM did not imply an absence of virus persistence. This finding raised at least two relevant questions. The first is whether BALB/b.*Cmv*1^r^ mice in which ECTV-IFN-α/βbp^Δ^ was below the limit of detection at 35 days p.i. (Fig 5H) had completely cleared virus. The second is whether virus might persist in disease-resistant C57BL/6 mice, in which virus genomes were below the limit of detection at day 35 p.i. (Fig 1H). This strain is known to effectively control ECTV infection and shows no evidence of a persistent infection.

Intriguingly, infectious virus was isolated from the BM, spleen and liver of BALB/b.*Cmv*1^r^ mice that had been infected with 10^5^ PFU ECTV-IFN-α/βbp^Δ^ and treated with CTX 80 days later (Fig 6E). Virus genomes were below the limit of detection at day 35 p.i. in this strain (Fig 5H), but sustained immunosuppression resulted in virus recrudescence. In contrast to ECTV-IFN-α/βbp^Δ^, we were unable to demonstrate the presence of infectious virus in BALB/c WT mice infected 80 days previously with 10^5^ PFU of the triple mutant ECTV-IFN-γbp^Δ^-IL-18bp^Δ^-SPI-2^Δ^ or ECTV-TK^Δ^ and subjected to immunosuppression over 4 weeks (S9A and S9B Fig).

The most unexpected but highly significant finding was made with the ECTV-resistant C57BL/6 strain. Infectious virus was isolated from the BM, spleen and liver of C57BL/6NCrl mice following treatment with CTX at 80 days p.i. (Fig 6F). The experiment was repeated using C57BL/6J mice to determine whether our results might be unique to the C57BL/NCrl mice. The C57BL/6J mice had been infected for more than 80 days with ECTV-WT and CTX treatment resulted in the death of one animal (Fig 6G) with severe liver necrosis and high titers of virus in this and other organs (S9C Fig), a hallmark of mousepox. Virus was not recovered from the remaining 3 animals sacrificed at 35 days post commencement of treatment. Taken together, the data established that ECTV persists in both resistant and susceptible strains of mice even though they recover from the infection. However, our results also indicate that highly attenuated strains of ECTV do not persist even in the susceptible strain of mice.

### CTX-treated mice transmit virus to co-housed naïve mice

**Fig 7:**
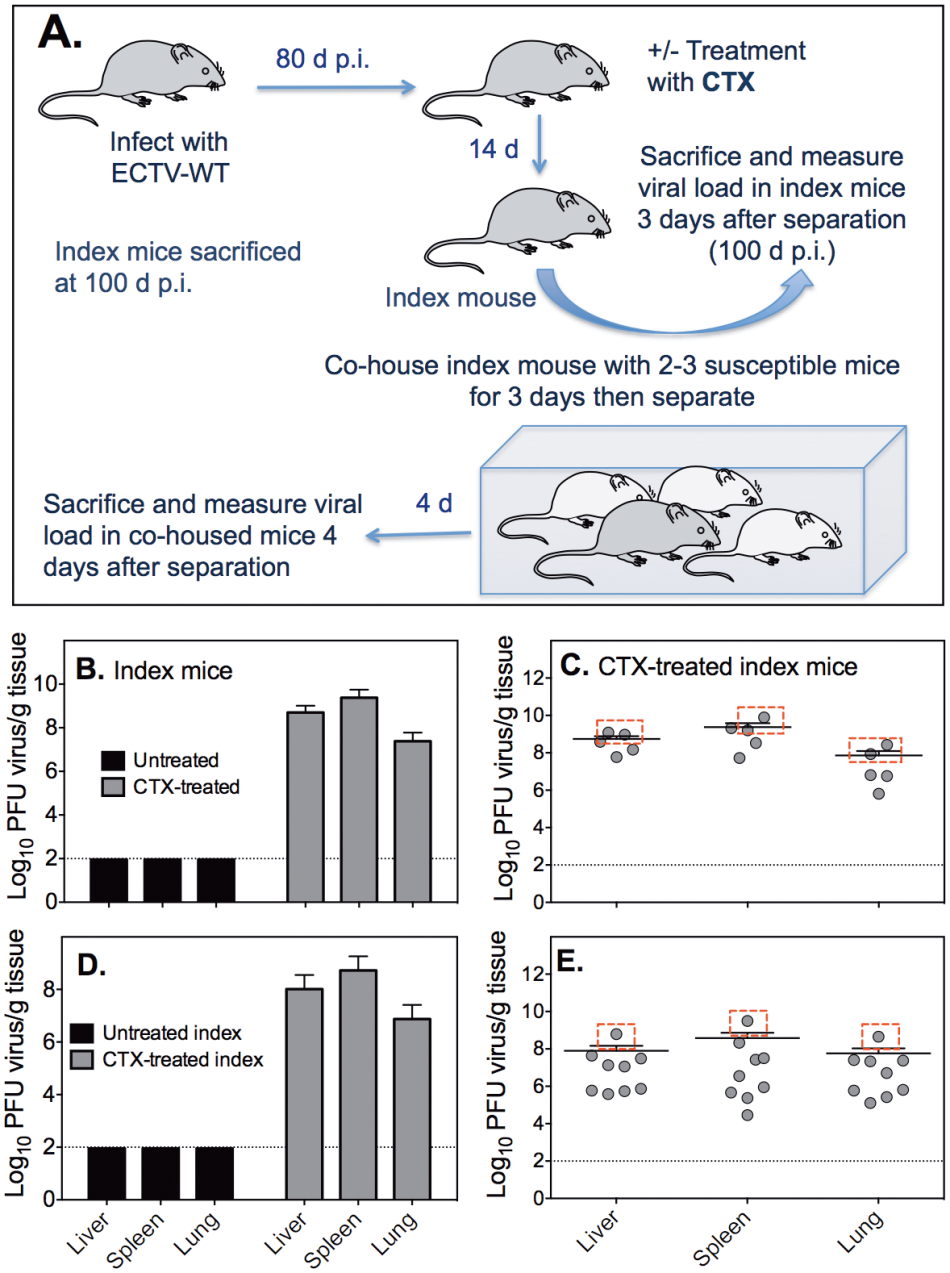
ECTV is transmitted from virus-infected, CTX-treated mice to naïve, co-housed mice. (A) Experimental scheme. Groups of BALB/c (index) mice were infected with 50 PFU ECTV-WT, rested for 80 days and treated with CTX or left untreated. (B) ECTV-WT titers in organs of untreated or CTX-treated index mice at 21 days post-commencement of treatment (101 days p.i.). (C) Viral load in organs of individual CTX-treated mice at 101 days p.i. (D) ECTV-WT titers in organs of naïve mice co-housed with untreated or CTX-treated mice. (E) Viral load in organs of individual naïve mice 4 days post-separation from index mice. For C and E, the boxed data shows viral load in organs of mice that succumbed to mousepox.

It is noteworthy that infectious virus was isolated from the BM of some mice at 101 days p.i. without immunosuppression (Fig 6B and 6C). We assessed whether similarly infected but untreated mice or CTX-treated mice that harbored high titers of ECTV could transmit virus to co-housed naïve animals. Groups of BALB/c (index) mice infected with a sub-lethal dose of ECTV-WT were rested for 80 days (Fig 7A). One group of index mice was treated with CTX and the second group was left untreated. Two weeks later, each treated or untreated index mouse was co-housed separately with 2–3 naïve animals for 3 days after which the latter were removed and housed in separate cages. Index mice were sacrificed 3 days after separation due the death of 2 animals. The co-housed naïve mice were sacrificed 4 days after separation when one animal succumbed to disease.

Infectious virus was not detectable in liver, spleen or lungs of index mice that had not been treated with CTX (Fig 7B). Consistent with the preceding data (Fig 6), CTX-treated mice harbored high titers of virus in all 3 organs. Two of the 5 index mice that died at 6 days post-co-housing harbored high titers of virus (Fig 7C, boxed data) but virus was not isolated from organs of mice co-housed with non-treated index mice (Fig 7D). In contrast, animals that were housed with CTX-treated index mice harbored virus (Fig 7D) of varying titers (Fig 7E). The one co-housed naïve mouse that died on day 7 post-exposure to a CTX-treated index mouse had the highest viral load in all organs (Fig 7E, boxed data). The data established that although infectious virus may be present at low levels in the BM of some animals several months p.i., they do not transmit virus. However, under conditions of immunosuppression, virus replicates to high titers and is able to be transmitted to co-housed naïve animals.

## Discussion

An acute viral infection can result in complete recovery of the host with or without residual sequelae, death or establishment of virus persistence [47]. Numerous mechanisms can potentially contribute to an acute viral infection becoming persistent but two factors thought to be critical are the effectiveness of host immune response in eliminating virus and subversion of the response by virus-encoded HRM. The OPV generally cause an acute infection in their hosts. However, some OPV, including ECTV, have been reported to persist in various animal species [13]. It has been suggested that this might be a reflection of persistent infection within a population rather than virus persistence in individual animals [13]. Our data indicate that the natural mouse pathogen ECTV can persist in the individual host.

Several lines of evidence indicate that ECTV causes a low-grade persistent infection in susceptible BALB/c and CBA strains of mice. First, virus genomes were detected in several organs but most consistently in the BM and blood several weeks after a primary infection. Although the virus load was below the limit of detection by viral plaque assay in most organs, infectious virus was nonetheless detected in a small proportion of animals in the BM at 101 days p.i. Second, virus persistence resulted in chronic stimulation of CD8 T cell responses, which were readily demonstrable *ex vivo*. ECTV-specific CD8 T cells were activated (CD62^lo^ and CD127^lo^) and functional as they were cytotoxic *ex vivo*, produced IFN-γ and their numbers (tetramer^+^) remained elevated throughout the course of the study. In contrast, in ECTV-resistant C57BL/6 mice the CTL activity is undetectable once virus is cleared [34]. Third, as seen in some models of persistent viral infections [48–52], a change in the immunodominance hierarchy of the CD8 T cell response was evident during the first 3 weeks of infection. Whether virus persistence is a cause or an effect of the switch in the immunodominance response is not known. Finally, the most definitive evidence for virus persistence was established through the use of the immunosuppressive drug CTX. Sustained immunosuppression over a 3-week period with CTX allowed ECTV to replicate to high titers and cause mousepox in all animals that had been infected 80–85 days previously. Such mice readily transmitted virus to co-housed naïve animals to cause disease.

Neither a single treatment with CTX nor the combined depletion of CD4 and CD8 T cell subsets, NK cells, granulocytes and plasmacytoid dendritic cells with monoclonal antibodies [46] every 2–3 days over a 3-week period resulted in virus recrudescence or signs of disease (S7 Fig). The requirement for sustained immunosuppression may be one reason why previous attempts to “reactivate” the disease in mice persistently infected with ECTV were unsuccessful [53]. It was evident that some animals not treated with CTX harboured low levels of virus in the BM but did not transmit virus to co-housed naïve mice. It is likely that virus was sequestered in the BM or another tissue below a threshold titer effective for transmission since animals that had recovered from infection, but not treated with CTX, did not transmit virus to cage mates. Previous reports indicate that ECTV can persist in sequestered sites of mice several weeks or months after sub-cutaneous infection without any shedding [1,2]. Virus shedding can nonetheless occur for extended periods during chronic intestinal tract infection in mice exposed to virus through the oral [3] or intra-gastric routes [4] but transmission does not occur beyond 36 days despite the presence of virus in the spleen of some animals at 95 days p.i [4]. The oral route of virus transmission can occur through cannibalism. We postulate that virus seeds and persists in the BM when viral load in visceral organs reaches a certain threshold. Further, in animals with a persistent infection, viral load in the BM or any other site needs to be above a certain threshold in order to seed visceral organs and be transmitted to other animals. Our ability to induce virus recrudescence through immunosuppression indicates that the immune system must keep virus under check and sequestered in tissues such as the BM.

Our results support a role for virus-encoded HRM in contributing to virus persistence. ECTV-encoded vIFN-γbp, vIL-18bp, SPI-2 and vIFN-α/βbp are known to down-regulate NK cell [38–41] and CTL [38,39] responses, both of which are critical for recovery of mice from infection. Most of these HRM also diminish IFN-γ production, which is critical for recovery of mice from mousepox [29,54]. Through dampening the host immune response, virus-encoded HRM impede effective virus clearance rapidly and as a consequence contribute to virus persistence. Deletion of gene(s) encoding one or two HRM reduced viral load in the liver early during the course of infection but was insufficient to overcome virus persistence. The most profound effect was evident in mice infected with the triple mutant (vIFN-γbp, vIL-18bp and SPI-2) or with ECTV-IFN-α/βbp^Δ^. Both viruses were cleared by day 7 in the liver and below the limit of detection in the blood and BM at day 35. However, when the inoculum dose was increased by 100- to 1,000-fold (10^4^−10^5^ PFU), ECTV-IFN-α/βbp^Δ^ was not cleared in the liver at day 7 and virus genomes persisted in the BM at day 35 p.i. In contrast, the highly attenuated TK mutant virus was below the limit of detection in mice infected with 10^6^ PFU. This data suggested that effective virus control early during the course of infection could overcome ECTV persistence. Accordingly, CTX treatment of BALB/c WT mice infected with high doses of ECTV-TK^Δ^ or the triple mutant did not result in recrudescence of virus. This was not the case with ECTV-IFN-α/βbp^Δ^ (discussed below), lending support to the idea that the combined actions of several HRM increase the propensity of ECTV to persist and in their absence virus is effectively cleared and does not persist.

Resistance to mousepox in C57BL/6 mice is associated with the capacity of this strain to generate robust innate and adaptive immunity [28–37,55], whereas these responses are sub-optimal in susceptible strains like BALB/c [33]. There are 4 known genetic loci in the C57BL/6 mouse genome that confer resistance to mousepox [27] whereas the BALB/c strain lacks alleles associated with resistance. In this study, BALB congenic strains that harbour resistance alleles at *Rmp-1* or *Rmp-3* were better able to control ECTV-WT replication and significantly reduce the numbers of virus genomes in the BM. In the BALB/b.*Cmv*1^r^ mice, which encodes both *Rmp-1* and *Rmp-3*, viral genomes were below the limit of detection, demonstrating that the combined contributions of resistance alleles at both loci were far more effective in virus control. This congenic strain also effectively controlled a high dose of the attenuated ECTV-IFN-α/βbp^Δ^ unlike the BALB/c WT mice, signifying that both host and viral factors impact on virus persistence. Nonetheless, despite our inability to demonstrate the presence of viral genomes in the BM of BALB/b.*Cmv*1^r^ mice infected with ECTV-IFN-α/βbp^Δ^, CTX treatment resulted in high titers of virus in organs. This finding further established that absence of viral genomes in the BM did not necessarily indicate complete virus clearance by the host and is consistent with the data obtained with C57BL/6 mice (discussed below).

We have investigated the roles of only two of four known loci that confer resistance to ECTV using the BALB congenic mice. It is conceivable that *Rmp-2*, which maps to the C5 complement component and *Rmp-4*, which maps to the selectin gene complex contribute to early virus control and potentially overcome persistence. In this regard, the C57BL/6 strain, which has alleles associated with resistance at all four loci is known to effectively clear virus with no evidence of a persistent infection. The fact that we were able to demonstrate recrudescence of virus and disease in 2 different lines of C57BL/6 mice several weeks after primary infection through immunosuppression indicates that ECTV can persist in a genetically resistant host that is immune without any clinical signs of disease or persistent infection. We do not know the mechanism(s) through which this happens or in what form(s) and where virus persists. These are fundamental questions that will need to be investigated. ECTV has been shown to persist for prolonged periods in a number of myeloma, lymphoma and hybridoma lines derived from ECTV-resistant or -susceptible mouse strains *in vitro* with no obvious adverse effects on the cells [56]. Further, the virus has been shown to persist in splenic dendritic cells and macrophages in BALB/c mice. These studies suggest that the virus might have a propensity to persist in certain types of cells in the BM of infected animals. Nonetheless, our finding raises the important question of whether persistence of ECTV in genetically resistant mice that are immune is unique to this virus model or if it is more widespread. There is at least one other example of persistence of virus that is generally associated with causing an acute infection in mice. Low levels of lymphocytic choriomeningitis virus (LCMV) strain WE have been shown to persist in immune C57BL/6 mice several weeks after an acute infection [57]. The authors suggest that virus persistence may in fact contribute to the maintenance of immunological memory. Although we do not have any direct evidence that ECTV persistence is necessary for maintenance of immunological memory, ECTV-WT induces a far more superior antibody response, which is life-long in C57BL/6 mice, whereas the response induced by ECTV-TK^Δ^ wanes rapidly [33,46,54,58–60]. In the current study, we have found that ECTV-WT can persist in C57BL/6 mice whereas ECTV-TK^Δ^ does not, even in the susceptible BALB/c strain.

Prolonged persistence of viruses (or viral nucleic acids) that cause either acute or chronic infections has been reported in humans, although in some cases persistence may be due to other underlying immunological conditions. West Nile virus has been found to persist in renal tissues of infected patients, either with chronic clinical symptoms or no symptoms, for more than 6 years [61,62]. Although measles virus is considered a prototype for viruses that cause acute infections, more recent studies indicate that viral RNA can persist in naturally infected children for months [63], significantly longer than previously thought. It has been suggested that cell-mediated immunity is involved in initial virus control and that the antibody response eventually clears measles viral RNA and prevents recurrent production of infectious virus [64] but it is not clear how long after infection this occurs. Finally, cryptic or occult infections with Hepatitis C virus (HCV) have been reported in some individuals. Occult HCV infection is characterized by the presence of viral RNA in the liver but in the absence of anti-HCV antibodies or HCV RNA in serum [65–67]. The underlying mechanisms of occult HCV are not fully understood but are believed to be multifactorial, including viral and host factors and co-infection with other pathogens. It is possible that HCV might be sequestered and replicates at low levels in immune privileged extra-hepatic sites, one consequence of which is the inability of the host to generate appropriate antiviral immunity. In individuals with occult HCV infections, virus reactivation might be expected to occur under conditions of chemotherapy or immunosuppression.

The requirement for cell-mediated immunity for early virus control and virus-specific antibody for complete virus clearance is well established for the mousepox model [33,34,55]. In C57BL/6 mice, ECTV infection becomes persistent in the absence of CD4 T cell-dependent antibody responses even in the presence of effector CD8 T-cell responses [32,34]. Hence, the role of antiviral antibody in overcoming a persistent infection in the BALB/c strain merits discussion. We have specifically only investigated the role of cell-mediated immunity in BALB/c mice but there is no question that an effective antibody response will be critical to overcoming virus persistence. Although BALB/c mice that survive an infection with ECTV-IFN-γbp^Δ^ generate strong antibody responses [38], virus still persists, suggesting the possibility that the response may not be effective. It is evident that antibody-mediated effector mechanisms can become defective during a persistent viral infection. High levels of viral antigen-antibody complexes are generated during a persistent LCMV infection, and these have been shown to suppress Fcγ-receptor-mediated antibody effector function [68,69]. A similar defect may be operative in ECTV-infected BALB/c mice, further contributing to virus persistence. Nonetheless, in the chronic LCMV model, the viral load is high and the antiviral CD8 T cells are exhausted [70]. This does not appear to be the case with ECTV infection of BALB/c mice, in which the viral load was very low and effector CD8 T cells were demonstrable throughout the entire period of study.

In summary, we have provided compelling evidence that ECTV causes a persistent infection in some susceptible strains of mice. The results lend support to previous reports of MPXV [8], CPXV [8–10] and VACV [11,12] persistence, possibly at a population level, in a variety of animal species. We have found that virus can persist in individual animals. Our finding that virus recrudescence can occur in ECTV-resistant C57BL/6 mice following sustained immunosuppression was unexpected. Nonetheless, these results in inbred strains of mice might be relevant and have implications for virus-host ecology and virus circulation in wild populations of mice. We speculate that wild mice could be subjected to stress and immunosuppression as a consequence of either food shortages during mouse plagues or during natural disasters. The occurrence of virus recrudescence in immunosuppressed mice under those situations may be rare but could potentially lead to virus spread, including through cannibalism, to naive animals. Whether immunosuppression caused under those conditions is equivalent to immunosuppression induced by treatment with CTX is not clear but further work is needed to understand the significance of virus persistence in resistant mice.

## Methods

### Ethics statement

This study was performed in strict accordance with the recommendations in the Australian code of practice for the care and use of animals for scientific purposes and the Australian National Health and Medical Research Council Guidelines and Policies on Animal Ethics. The Australian National University Animal Ethics and Experimentation Committee approved all animal experiments (Protocol Numbers: J.IG.75.09 and A2012/041). Tribromoethanol (Avertin) was used as the anesthetic (200–240 mg/kg body weight) given via intra-peritoneal injection prior to infection with virus. The respiration rate of the animals was monitored during anesthesia and recovery took place upon a warm table. Animals were euthanized by cervical dislocation.

### Mice

Inbred, specific-pathogen-free female BALB/c (H-2^d^), BALB/b (H-2^b^), CBA/H (H-2^k^), C57BL/6 mice and BALB congenic strains C.B6-*Klra8* ^*Cmv1-r*^/UwaJ (BALB/c.*Cmv1* ^r^) [45] and B.B6-*Klra8* ^*Cmv1-r*^/UwaJ (BALB/b.*Cmv*1^r^) were bred at the ANU Bioscience Services. The congenic strains carry C57BL/6 alleles in the NKC on mouse chromosome 6 including NK1.1. The NKC in the BALB strain lacks some activating receptors and is a known locus for resistance to ECTV. Mice were used at 6–10 weeks of age. The highly susceptible A/J strain was purchased from Animal Resources Centre, Western Australia and used in virus transmission experiments.

### Cell lines and cell cultures

BS-C-1 (ATCC CCL-26), epithelial kidney cell line from African green monkey, CV-1 cells (ATCC CCL-70), fibroblast kidney cell line from African green monkey, P-815 (H-2^d^; ATCC TIB-64), a DBA/2 mouse-derived mastocytoma and MC57G (H-2^b^; ATCC CRL-2295), a C57BL/6J mouse-derived fibrosarcoma, were obtained from American Type Culture Collection (Rockville, MD). All cells were maintained in Eagle’s Minimum Essential Medium (GIBCO) supplemented with 10% fetal calf serum (Sigma-Aldrich Inc., St. Louis MO, USA), 2mM L-glutamine (GIBCO), 120 μg/ml penicillin and 200μg/ml streptomycin and neomycin sulfate (GIBCO).

### Viruses and infection

The Moscow strain of wild type ECTV (ATCC VR1374), designated ECTV-WT and the vIFN-γ bp deletion mutant virus derived from ECTV-WT, designated ECTV-IFN-γbp^Δ^ [38] were used. In addition, ECTV deletion mutant viruses lacking vIFN-α/βbp (ECTV-IFN-α/βbp^Δ^), vIL-18bp (ECTV-IL-18bp^Δ^), serine protease inhibitor 2 (ECTV-SPI-2^Δ^), vIFN-γbp and IL-18bp (ECTV-IFN-γbp^Δ^-IL-18bp^Δ^; double mutant), vIFN-γbp, IL-18bp and SPI-2 (ECTV-IFN-γbp^Δ^-IL-18bp^Δ^-SPI-2^Δ^; triple mutant) and thymidine kinase (ECTV-TK^Δ^) [71] were used. The mutant viruses were generated as described elsewhere [38]. All ECTV strains were propagated in BS-C-1 cells, titrated using viral plaque assay (VPA) [32] and all mutant viruses were found to replicate to levels comparable with ECTV-WT (S10 Fig). Mice were inoculated with 50, 100 or 500 PFU ECTV-WT, 100 or 500 PFU ECTV-IFN-γbp^Δ^ and 10^5^ or 10^6^ PFU ECTV-TK^Δ^ subcutaneously (s.c.) in the flank of the left hind leg (hock) under avertin anesthesia. In all animal experiments, a back titration of the virus inoculum was performed routinely to ensure consistency and the correct dose was used.

### CTX treatment and virus transmission experiments

The use of CTX in experiments was authorized under the Work Health and Safety Act of 2011, Australia. Mice that had been infected with ECTV 80 days previously were injected with 240 mg/kg CTX (Sigma-Aldrich) through the intra-peritoneal route every 5 days, with a total of 3 injections over a period of 15 days and observed for a further 6–7 days. The ECTV-resistant C57BL/6 strains were similarly treated but with a total of 4 injections over a period of 20 days and observed for a further 7–15 days.

For virus transmission experiments, some index BALB/c mice were treated three times with CTX whereas controls were left untreated. Two weeks later, each treated or untreated index mouse was co-housed separately with 2–3 naïve A/J mice for 3 days after which the latter were removed and housed in separate cages. Index mice were sacrificed 3 days after separation due the death of 2 animals. The co-housed naïve mice were sacrificed 4 days after separation when one animal succumbed to disease.

### Combined leukocyte cell subset depletion to induce immunosuppression

As an alternative to CTX, multiple leukocyte subsets were depleted to induce immunosuppression in mice. WT C57BL/6 mice infected with 1000 PFU of ECTV-WT 80 days previously were treated with monoclonal antibodies to deplete granulocytes (clone RB6-8C5), plasmacytoid dendritic cells (clone 120G8), NK cells (clone PK136), CD4 T cells (clone GK1.5) and CD8 T cells (clone 2.43.1) as described previously [46] every 2–3 days for 3 weeks and sacrificed to measure viral load in organs.

### Synthetic peptides

ECTV-specific CD8 T cell determinants restricted by H-2d L^d^-EVM026_26–34_ (L^d^-026), K^d^-EVM149.5_44–52_ (K^d^-149.5) and D^d^-EVM043_140-148_ (D^d^-043) [72] used in this study are shown in S1 Table. Peptides were synthesized and purified via reverse-phase HPLC at the BRF, JCSMR, Australian National University.

### Cytolytic T lymphocyte assays

Direct *ex vivo* cytolytic activity of splenic CTL was determined at various effector-to-target ratios in 6-hr standard ^51^Chromium (^51^Cr)-release assays as described elsewhere [33]. Briefly, spleen cells from infected animals were assessed for their ability to kill ^51^Cr-labeled ECTV-infected, ECTV peptide determinant-pulsed or uninfected syngeneic P815 (H-2^d^) or MC57G (H-2^b^) targets. The ECTV peptide determinants used are listed in S1 Table.

### Intracellular cytokine staining (ICS) for IFN-γ

Antigen-specific IFN-γ producing CD8 T cells were enumerated using intracellular cytokine staining as described elsewhere [72] using anti-CD8α-APC (clone 53–6.7) and anti-IFN-γ-PE (clone XMG1.2) (BD Biosciences). Total events for cells were acquired using a FACSCalibur flow cytometer (BD Biosciences) and analyzed using FlowJo software (Tree Star Inc.).

### Peptide-MHC class I tetramer and Vβ TCR chain usage analysis

Tetrameric H-2^d^ MHC class I complexes folded with L^d^-026, K^d^-149.5 and D^d^-043 peptides (S1 Table), were used to phenotype determinant-specific CD8 T cells. Spleen cells were stained with PE-conjugated peptide-MHC class I tetramers and anti-CD8α-APC at 4°C for 60 min and subsequently washed twice with PBS containing 2% FCS before analysis. For analysis of Vβ TCR chain usage, splenocytes were stained with anti-CD8α-APC, anti-Vβ TCR-FITC screening panel of antibodies (BD Biosciences) and PE-conjugated MHC class I tetramers. Data was acquired on a LSR Fortessa flow cytometer (BD Biosciences) with BD FACS Diva software and analyzed using FlowJo Software (Tree Star, Inc).

### Determination of viral load

Tissue removed aseptically from mice was stored at -80°C until processed. Virus titers, expressed as log10 PFU/gram tissue were determined on BSC-1 monolayers using the conventional viral plaque assay, as described previously [32,33,73]. Briefly, organs were weighed and homogenized in 1 ml PBS, dispersed by sonication and used to make serial 10-fold dilutions. A 100μl volume of the homogenate was plated onto monolayers of B-SC-1 cells beginning at 10^−1^ dilution. Undiluted organ homogenates were toxic the B-SC-1 monolayers and were not used. The limit of detection of virus was therefore 100 PFU. Viral load below the limit of detection by viral plaque assay was measured by quantitative real time PCR (qRT-PCR), as described elsewhere [46] to amplify the target sequence of the late gene ECTV-Mos-156 that encodes the viral hemagglutinin. The limit of detection of viral genomes by qRT-PCR is 10 copies. One PFU of ECTV-WT is equivalent to 275 genome copies. Genome copy numbers above 10 are considered biologically significant, as the LD_50_ of the highly susceptible A/J mouse strain to ECTV-WT is 0.04 PFU, i.e. 11 genome copies.

### Statistical analysis

Statistical analyses of experimental data, employing parametric and nonparametric tests as indicated, were performed using GraphPad Prism (GraphPad Software, San Diego USA).

## Supporting Information

S1 Fig CD8 T cell responses during ECTV-WT or ECTV-TK^Δ^ infection. Groups of female BALB/c mice were infected with 500 PFU ECTV-WT or 2 x 10^6^ PFU ECTV-TK^Δ^ s.c., sacrificed on day 7 p.i. and splenic CTL activity was measured. Percent specific lysis of virus-infected (Total) or ECTV CD8 T cell determinant-pulsed, ^51^Cr-labelled P815 targets by splenocytes from ECTV-WT-infected (A) or ECTV-TK^Δ^-infected (B) mice.
(TIFF)

S2 Fig Change in immunodominance hierarchy of CD8 T cell responses in BALB/c mice infected with a sub-lethal dose of ECTV-WT. BALB/c mice were infected with 100 PFU of ECTV-WT or 500 PFU of ECTV-IFN-γbp^Δ^ and splenocytes were used to measure CD8 T cell responses at the days indicated. (A) Numbers of peptide-MHC class I tetramer^+^ CD8 T cells ± SEM at days 7, 14 and 21 p.i. with ECTV-WT. (B) *Ex vivo* cytolytic activity of splenocytes from ECTV-WT-infected mice 21 days p.i. against ECTV-infected (total) or ECTV peptide determinant-pulsed P815 target cells. *P* values were obtained by Mann-Whitney U test for the indicated comparisons (A and B): *, P<0.05; **, P<0.01; ***, P<0.001. (C) Numbers of peptide-MHC class I tetramer^+^ CD8 T cells in BALB/c mice 21 p.i. with 100 PFU ECTV-WT or 500 PFU ECTV-IFN-γbp^Δ^.
(TIFF)

S3 Fig Vβ TCR chain usage by CD8 T cell determinant-specific primary anti-ECTV effector T cells. Groups of 6 BALB/c mice were infected with 500 PFU ECTV-IFN-γbp^Δ^. On the days indicated, mice were sacrificed and splenocytes were co-stained for CD8α (anti-CD8α-APC), mouse VβTCR phenotypes (anti-Vβ TCR-FITC screening panel) and PE-conjugated H-2^d^ tetramers. Events were gated on CD8 T cells and proportions of tetramer-positive CD8 T cells expressing specific Vβ TCR over three weeks p.i. with ECTV-IFN-γbp^Δ^ is shown. Vβ TCR usage at (A) day 7, (B) day 14 and (C) day 21 p.i. Data shown are means ± SEM of two separate animal experiments of 6 mice per group.
(TIFF)

S4 Fig Persistence of activated, ECTV-specific CD8 T cells at day 37. Groups of 5 BALB/c mice were infected with ECTV-IFN-γbp^Δ^ and sacrificed at days 0 (naïve), 7, 14, 21 and 37 p.i.. (A) Relative proportions of CD62L^hi^ and CD127^hi^ CD8 T cells during early and late primary immune response. (B) Numbers of ECTV-specific (total) and ECTV peptide determinant-specific IFN-γ^+^ CD8 T cells at day 37 p.i. (C) *Ex vivo* cytolytic activity of splenocytes from ECTV-IFN-γbp^Δ^ infected mice 37 days p.i. against ECTV-infected (total) or ECTV peptide determinant-pulsed P815 target cells. (D) Numbers of peptide-MHC class I tetramer^+^ CD8 T cells at day 37 p.i. *P* values were obtained by Mann-Whitney U test for the indicated comparisons (B, C and D): *, *P*<0.05; **, *P*<0.01.
(TIFF)

S5 Fig Kinetic analyses of tetramer+ or IFN-γ+ CD8 T cell proportions generated by infection with ECTV-IFN-γbpΔ. Data on CD8 T cell proportions presented in this figure are derived from the same experiment for which some results are presented in Fig 4. Groups of 6 BALB/c mice were infected with 500 PFU ECTV-IFN-γbp^Δ^ and phenotypic assays undertaken on the days indicated. (A) Kinetics of ECTV-specific (total) IFN-γ^+^ CD8 T cell proportions, with or without stimulation with ECTV. (B) Kinetics of ECTV peptide determinant-specific IFN-γ^+^ CD8 T cell proportions. (C) Kinetics of peptide-MHC class I tetramer^+^ CD8 T cell proportions from day 7 till day 51.
(TIFF)

S6 Fig Virus-encoded HRM and host resistance loci mapping to the NKC and MHC impact on virus persistence. Data presented in this figure is derived from the same experiment for which some results are presented in Fig 5. Groups of 5 female BALB/c mice were infected and sacrificed on day 35 to measure viral load. (A) Virus genome copy numbers in blood of BALB/c mice 35 days p.i. with 100 PFU of WT, single mutant, double mutant or triple mutant ECTV. The limit of virus genome detection is 10 copies and is shown by the dotted line. (B) Viral load in the liver at day 7 p.i. in BALB/c mice infected with varying doses of ECTV-IFN-α/βbp^Δ^ or ECTV-TK^Δ^. The limit of virus detection is 2 log_10_ PFU, shown by the dotted line. (C) Depletion of NK cells in the congenic BALB/c.*Cmv*1^r^ and BALB/b.*Cmv*1^r^ mice by treatment with anti-NK1.1 mAb overcomes resistance to ECTV-WT infection. The congenic strains but not WT BALB/c or BALB/b mice express the NK1.1 antigen. *P* values for (A) and (B) were obtained by Mann-Whitney U test: *P<0.05, **P<0.01, and ***P<0.001. *P* values in (C) were obtained by Log-rank (Mantel-Cox) test: **, P<0.01.
(TIFF)

S7 Fig Viral load in organs of C57BL/6 mice treated with leukocyte depleting monoclonal antibodies. Groups of 5 WT C57BL/6 mice were infected with 1000 PFU of ECTV-WT. Beginning at 80 days p.i, mice were treated with monoclonal antibodies every 2–3 days to deplete NK cells, granulocytes, plasmacytoid dendritic cells, CD4 T cells and CD8 T cells for a period of 3 weeks. Viral load was measured in the indicated organs 3 days after the last treatment (day 104 p.i).
(TIFF)

S8 Fig Virus genomes in organs of BALB/c mice at 80 days p.i. Data presented in this figure is derived from the same experiment for which some results are presented in Fig 6. Groups of 5 WT BALB/c mice were infected with 100 PFU of ECTV-WT or ECTV-IFN-γbp^Δ^ and sacrificed at day 80 p.i. to quantify virus genomes in various organs. Virus genome copy numbers in organs of BALB/c mice infected with (A) ECTV-WT or (B) ECTV-IFN-γbp^Δ^.
(TIFF)

S9 Fig Immunosuppression with CTX and virus recrudescence. For A and B, groups of 5 WT BALB/c mice were infected with 10^5^ PFU of ECTV-TK^Δ^ or the triple mutant ECTV-IFN-γbp^Δ^-IL-18bp^Δ^-SPI-2^Δ^ and subjected to immunosuppression with CTX over 4 weeks. Shown are titers of (A) ECTV-TK^Δ^ and (B) ECTV-IFN-γbp^Δ^-IL-18bp^Δ^-SPI-2^Δ^ in the various organs. For C, organs from the one C57BL/6J mice infected for over 80 days with 10^3^ PFU ECTV-WT and treated with CTX that died were collected for determination of viral load. Shown are virus titers in the BM, liver, spleen and lung (C).
(TIFF)

S10 Fig The *in vitro* replicative capacity of WT and mutant viruses. Monolayers of BS-C-1 cells were infected with WT or single mutant viruses (A) or WT, double or triple mutant viruses (B) at 0.1 PFU/ cell in 12-well plates. Cells and supernatant were harvested at the indicated times and the viral load measured by virus plaque assay. Data shown are means ± SD of triplicate cultures.
(TIFF)

S1 Table MHC class I H-2d peptide determinants. MHC restricting elements, nomenclature and linear sequence of MHC class I H-2d peptide determinants from ECTV used to measure determinant-specific CTL responses, intracellular IFN-γ expression and to generate MHC class I-peptide tetramers used in this study.
(EPS)

## References

[1] Fenner F (1948) The epizootic behaviour of mouse-pox (infectious ectromelia of mice) the course of events in long-continued epidemics. J Hyg (Lond) 46: 383–393. 18129315 10.1017/s002217240003655x PMC2235147

[2] Fenner F (1948) The epizootic behaviour of mouse-pox, infectious ectromelia. Br J Exp Pathol 29: 69–91. 18865106 PMC2073073

[3] Gledhill AW (1962) Latent ectromelia. Nature 196: 298 13948424 10.1038/196298a0

[4] Wallace GD, Buller RM (1985) Kinetics of ectromelia virus (mousepox) transmission and clinical response in C57BL/6J, BALB/cByJ and AKR/J inbred mice. Lab Anim Sci 35: 41–46. 2984458

[5] Wallace GD, Buller RM, Morse HC 3rd (1985) Genetic determinants of resistance to ectromelia (mousepox) virus-induced mortality. J Virol 55: 890–891. 2991610 10.1128/jvi.55.3.890-891.1985 PMC255083

[6] de Faundez IM, Gierynska M., Niemialtowski M.G., Malicka E, and Popis A., editor (1995) Ectromelia Virus Establishes a Persistent Infection in Spleen Dendritic Cells and Macrophages of BALB/c Mice following the Acute Disease. New York: Springer US pp 257–261 p. 10.1007/978-1-4615-1971-3_57 8526068

[7] Toka FN, Spohr C. I., Schollenberger A., Krzyzanowska M., Gierynska M., Ru M. and Niemialtowski M (2005) Ectromelia virus persistence revisited: virus detection by in situ PCR after long-term infection of BALB/c mice. Central European Journal of Immunology 30: 17–25.

[8] Shelukhina EM, Shenkman LS, Rozina EE, Marennikova SS (1979) [Possible mechanism of orthopoxvirus preservation in nature]. Vopr Virusol: 368–372. 225883

[9] Marennikova SS, Ladnyj ID, Ogorodinikova ZI, Shelukhina EM, Maltseva NN (1978) Identification and study of a poxvirus isolated from wild rodents in Turkmenia. Arch Virol 56: 7–14. 204271 10.1007/BF01317279

[10] Maiboroda AD (1982) Experimental infection of Norvegian rats (Rattus norvegicus) with ratpox virus. Acta Virol 26: 288–291. 6127938

[11] Olitsky PK, Long PH (1929) Relation of vaccinal immunity to the persistence of the virus in rabbits J Exp Med 50: 263–272. 19869620 10.1084/jem.50.3.263 PMC2131631

[12] Ginsberg AH, Johnson KP (1977) The effect of cyclophosphamide on intracerebral vaccinia virus infection in Balb/C mice. Exp Mol Pathol 27: 285–294. 923746 10.1016/0014-4800(77)90001-6

[13] Fenner F. H DA, Arita I., Jezek Z., Ladnyi I.D. (1988) Smallpox and its Eradication. Geneva: World Health Organization.

[14] Henderson DA (1998) Bioterrorism as a public health threat. Emerg Infect Dis 4: 488–492. 9716981 10.3201/eid0403.980340 PMC2640310

[15] Arita I, Jezek Z, Khodakevich L, Ruti K (1985) Human monkeypox: a newly emerged orthopoxvirus zoonosis in the tropical rain forests of Africa. Am J Trop Med Hyg 34: 781–789. 2992305 10.4269/ajtmh.1985.34.781

[16] Reynolds MG, Damon IK (2012) Outbreaks of human monkeypox after cessation of smallpox vaccination. Trends Microbiol 20: 80–87. 10.1016/j.tim.2011.12.001 22239910

[17] Parker S, Nuara A, Buller RM, Schultz DA (2007) Human monkeypox: an emerging zoonotic disease. Future Microbiol 2: 17–34. 17661673 10.2217/17460913.2.1.17

[18] de Assis FL, Vinhote WM, Barbosa JD, de Oliveira CH, de Oliveira CM, et al (2013) Reemergence of vaccinia virus during Zoonotic outbreak, Para State, Brazil. Emerg Infect Dis 19: 2017–2020. 10.3201/eid1912.130589 24274374 PMC3840876

[19] Kroon EG, Mota BE, Abrahao JS, da Fonseca FG, de Souza Trindade G (2011) Zoonotic Brazilian Vaccinia virus: from field to therapy. Antiviral Res 92: 150–163. 10.1016/j.antiviral.2011.08.018 21896287

[20] Vogel S, Sardy M, Glos K, Korting HC, Ruzicka T, et al (2012) The Munich outbreak of cutaneous cowpox infection: transmission by infected pet rats. Acta Derm Venereol 92: 126–131. 10.2340/00015555-1227 22041995

[21] Campe H, Zimmermann P, Glos K, Bayer M, Bergemann H, et al (2009) Cowpox virus transmission from pet rats to humans, Germany. Emerg Infect Dis 15: 777–780. 10.3201/eid1505.090159 19402967 PMC2687013

[22] Vorou RM, Papavassiliou VG, Pierroutsakos IN (2008) Cowpox virus infection: an emerging health threat. Curr Opin Infect Dis 21: 153–156. 10.1097/QCO.0b013e3282f44c74 18317038

[23] Di Giulio DB, Eckburg PB (2004) Human monkeypox: an emerging zoonosis. Lancet Infect Dis 4: 15–25. 14720564 10.1016/S1473-3099(03)00856-9 PMC9628772

[24] Reed KD, Melski JW, Graham MB, Regnery RL, Sotir MJ, et al (2004) The detection of monkeypox in humans in the Western Hemisphere. N Engl J Med 350: 342–350. 14736926 10.1056/NEJMoa032299

[25] Schell K (1960) Studies on the innate resistance of mice to infection with mousepox. II. Route of inoculation and resistance; and some observations on the inheritance of resistance. Aust J Exp Biol Med Sci 38: 289–299. 13747447 10.1038/icb.1960.30

[26] O'Neill HC, Blanden RV, O'Neill TJ (1983) H-2-linked control of resistance to ectromelia virus infection in B10 congenic mice. Immunogenetics 18: 255–265. 6311733 10.1007/BF00952964

[27] Esteban DJ, Buller RM (2005) Ectromelia virus: the causative agent of mousepox. J Gen Virol 86: 2645–2659. 16186218 10.1099/vir.0.81090-0

[28] Jacoby RO, Bhatt PN, Brownstein DG (1989) Evidence that NK cells and interferon are required for genetic resistance to lethal infection with ectromelia virus. Arch Virol 108: 49–58. 2480764 10.1007/BF01313742

[29] Karupiah G, Fredrickson TN, Holmes KL, Khairallah LH, Buller RM (1993) Importance of interferons in recovery from mousepox. J Virol 67: 4214–4226. 7685412 10.1128/jvi.67.7.4214-4226.1993 PMC237791

[30] Karupiah G, Xie QW, Buller RM, Nathan C, Duarte C, et al (1993) Inhibition of viral replication by interferon-gamma-induced nitric oxide synthase. Science 261: 1445–1448. 7690156 10.1126/science.7690156

[31] Ramshaw IA, Ramsay AJ, Karupiah G, Rolph MS, Mahalingam S, et al (1997) Cytokines and immunity to viral infections. Immunol Rev 159: 119–135. 9416507 10.1111/j.1600-065x.1997.tb01011.x

[32] Karupiah G, Buller RM, Van Rooijen N, Duarte CJ, Chen J (1996) Different roles for CD4+ and CD8+ T lymphocytes and macrophage subsets in the control of a generalized virus infection. J Virol 70: 8301–8309. 8970949 10.1128/jvi.70.12.8301-8309.1996 PMC190917

[33] Chaudhri G, Panchanathan V, Buller RM, van den Eertwegh AJ, Claassen E, et al (2004) Polarized type 1 cytokine response and cell-mediated immunity determine genetic resistance to mousepox. Proc Natl Acad Sci U S A 101: 9057–9062. 15184649 10.1073/pnas.0402949101 PMC428472

[34] Chaudhri G, Panchanathan V, Bluethmann H, Karupiah G (2006) Obligatory requirement for antibody in recovery from a primary poxvirus infection. J Virol 80: 6339–6344. 16775322 10.1128/JVI.00116-06 PMC1488964

[35] Parker AK, Parker S, Yokoyama WM, Corbett JA, Buller RM (2007) Induction of natural killer cell responses by ectromelia virus controls infection. J Virol 81: 4070–4079. 17287257 10.1128/JVI.02061-06 PMC1866162

[36] Moulton EA, Atkinson JP, Buller RM (2008) Surviving mousepox infection requires the complement system. PLoS Pathog 4: e1000249 10.1371/journal.ppat.1000249 19112490 PMC2597719

[37] Fang M, Lanier LL, Sigal LJ (2008) A role for NKG2D in NK cell-mediated resistance to poxvirus disease. PLoS Pathog 4: e30 10.1371/journal.ppat.0040030 18266471 PMC2233669

[38] Sakala IG, Chaudhri G, Buller RM, Nuara AA, Bai H, et al (2007) Poxvirus-encoded gamma interferon binding protein dampens the host immune response to infection. J Virol 81: 3346–3353. 17229697 10.1128/JVI.01927-06 PMC1866021

[39] Xu RH, Cohen M, Tang Y, Lazear E, Whitbeck JC, et al (2008) The orthopoxvirus type I IFN binding protein is essential for virulence and an effective target for vaccination. J Exp Med 205: 981–992. 10.1084/jem.20071854 18391063 PMC2292233

[40] Melo-Silva CR, Tscharke DC, Lobigs M, Koskinen A, Wong YC, et al (2011) The ectromelia virus SPI-2 protein causes lethal mousepox by preventing NK cell responses. J Virol 85: 11170–11182. 10.1128/JVI.00256-11 21849445 PMC3194934

[41] Born TL, Morrison LA, Esteban DJ, VandenBos T, Thebeau LG, et al (2000) A poxvirus protein that binds to and inactivates IL-18, and inhibits NK cell response. J Immunol 164: 3246–3254. 10706717 10.4049/jimmunol.164.6.3246

[42] Delano ML, Brownstein DG (1995) Innate resistance to lethal mousepox is genetically linked to the NK gene complex on chromosome 6 and correlates with early restriction of virus replication by cells with an NK phenotype. J Virol 69: 5875–5877. 7637035 10.1128/jvi.69.9.5875-5877.1995 PMC189465

[43] Brownstein DG, Bhatt PN, Gras L, Budris T (1992) Serial backcross analysis of genetic resistance to mousepox, using marker loci for Rmp-2 and Rmp-3. J Virol 66: 7073–7079. 1433507 10.1128/jvi.66.12.7073-7079.1992 PMC240377

[44] Fang M, Orr MT, Spee P, Egebjerg T, Lanier LL, et al (2011) CD94 is essential for NK cell-mediated resistance to a lethal viral disease. Immunity 34: 579–589. 10.1016/j.immuni.2011.02.015 21439856 PMC3081423

[45] Scalzo AA, Lyons PA, Fitzgerald NA, Forbes CA, Shellam GR (1995) The BALB.B6-Cmv1r mouse: a strain congenic for Cmv1 and the NK gene complex. Immunogenetics 41: 148–151. 7806288 10.1007/BF00182328

[46] Tahiliani V, Chaudhri G, Eldi P, Karupiah G (2013) The orchestrated functions of innate leukocytes and T cell subsets contribute to humoral immunity, virus control, and recovery from secondary poxvirus challenge. J Virol 87: 3852–3861. 10.1128/JVI.03038-12 23345522 PMC3624232

[47] Virgin HW, Wherry EJ, Ahmed R (2009) Redefining chronic viral infection. Cell 138: 30–50. 10.1016/j.cell.2009.06.036 19596234

[48] Bergmann CC, Altman JD, Hinton D, Stohlman SA (1999) Inverted immunodominance and impaired cytolytic function of CD8+ T cells during viral persistence in the central nervous system. J Immunol 163: 3379–3387. 10477608

[49] Obar JJ, Fuse S, Leung EK, Bellfy SC, Usherwood EJ (2006) Gammaherpesvirus persistence alters key CD8 T-cell memory characteristics and enhances antiviral protection. J Virol 80: 8303–8315. 16912282 10.1128/JVI.00237-06 PMC1563881

[50] Stevenson PG, Belz GT, Altman JD, Doherty PC (1999) Changing patterns of dominance in the CD8+ T cell response during acute and persistent murine gamma-herpesvirus infection. Eur J Immunol 29: 1059–1067. 10229071 10.1002/(SICI)1521-4141(199904)29:04<1059::AID-IMMU1059>3.0.CO;2-L

[51] Wherry EJ, Blattman JN, Murali-Krishna K, van der Most R, Ahmed R (2003) Viral persistence alters CD8 T-cell immunodominance and tissue distribution and results in distinct stages of functional impairment. J Virol 77: 4911–4927. 12663797 10.1128/JVI.77.8.4911-4927.2003 PMC152117

[52] van der Most RG, Murali-Krishna K, Lanier JG, Wherry EJ, Puglielli MT, et al (2003) Changing immunodominance patterns in antiviral CD8 T-cell responses after loss of epitope presentation or chronic antigenic stimulation. Virology 315: 93–102. 14592762 10.1016/j.virol.2003.07.001

[53] Gledhill AW (1962) The problems of laboratory animal disease In: H R.J.C., editor. vIRAL diseases in laboratory animals. New York: Academic Press pp. 99–112.

[54] Panchanathan V, Chaudhri G, Karupiah G (2006) Protective immunity against secondary poxvirus infection is dependent on antibody but not on CD4 or CD8 T-cell function. J Virol 80: 6333–6338. 16775321 10.1128/JVI.00115-06 PMC1488959

[55] Fang M, Sigal LJ (2005) Antibodies and CD8+ T cells are complementary and essential for natural resistance to a highly lethal cytopathic virus. J Immunol 175: 6829–6836. 16272340 10.4049/jimmunol.175.10.6829

[56] Buller RM, Weinblatt AC, Hamburger AW, Wallace GD (1987) Observations on the replication of ectromelia virus in mouse-derived cell lines: implications for epidemiology of mousepox. Lab Anim Sci 37: 28–32. 3035276

[57] Ciurea A, Klenerman P, Hunziker L, Horvath E, Odermatt B, et al (1999) Persistence of lymphocytic choriomeningitis virus at very low levels in immune mice. Proc Natl Acad Sci U S A 96: 11964–11969. 10518559 10.1073/pnas.96.21.11964 PMC18395

[58] Panchanathan V, Chaudhri G, Karupiah G (2005) Interferon function is not required for recovery from a secondary poxvirus infection. Proc Natl Acad Sci U S A 102: 12921–12926. 16123129 10.1073/pnas.0505180102 PMC1200282

[59] Panchanathan V, Chaudhri G, Karupiah G (2010) Antiviral protection following immunization correlates with humoral but not cell-mediated immunity. Immunol Cell Biol 88: 461–467. 10.1038/icb.2009.110 20066003

[60] Chaudhri G, Tahiliani V, Eldi P, Karupiah G (2015) Vaccine-induced protection against orthopoxvirus infection is mediated through the combined functions of CD4 T cell-dependent antibody and CD8 T cell responses. J Virol 89: 1889–1899. 10.1128/JVI.02572-14 25428875 PMC4300738

[61] Murray K, Walker C, Herrington E, Lewis JA, McCormick J, et al (2010) Persistent infection with West Nile virus years after initial infection. J Infect Dis 201: 2–4. 10.1086/648731 19961306 PMC2791189

[62] Gould EA (2010) West Nile virus: don't underestimate its persistence. J Infect Dis 201: 1 10.1086/648732 19961305

[63] Riddell MA, Moss WJ, Hauer D, Monze M, Griffin DE (2007) Slow clearance of measles virus RNA after acute infection. J Clin Virol 39: 312–317. 17625962 10.1016/j.jcv.2007.05.006

[64] Lin WH, Kouyos RD, Adams RJ, Grenfell BT, Griffin DE (2012) Prolonged persistence of measles virus RNA is characteristic of primary infection dynamics. Proc Natl Acad Sci U S A 109: 14989–14994. 10.1073/pnas.1211138109 22872860 PMC3443140

[65] Castillo I, Pardo M, Bartolome J, Ortiz-Movilla N, Rodriguez-Inigo E, et al (2004) Occult hepatitis C virus infection in patients in whom the etiology of persistently abnormal results of liver-function tests is unknown. J Infect Dis 189: 7–14. 14702147 10.1086/380202

[66] Lerat H, Hollinger FB (2004) Hepatitis C virus (HCV) occult infection or occult HCV RNA detection? J Infect Dis 189: 3–6. 14702146 10.1086/380203

[67] Attar BM, Van Thiel D (2015) A New Twist to a Chronic HCV Infection: Occult Hepatitis C. Gastroenterol Res Pract 2015: 579147 10.1155/2015/579147 26221136 PMC4495183

[68] Wieland A, Shashidharamurthy R, Kamphorst AO, Han JH, Aubert RD, et al (2015) Antibody effector functions mediated by Fcgamma-receptors are compromised during persistent viral infection. Immunity 42: 367–378. 10.1016/j.immuni.2015.01.009 25680276 PMC4339104

[69] Yamada DH, Elsaesser H, Lux A, Timmerman JM, Morrison SL, et al (2015) Suppression of Fcgamma-receptor-mediated antibody effector function during persistent viral infection. Immunity 42: 379–390. 10.1016/j.immuni.2015.01.005 25680277 PMC4334737

[70] Moskophidis D, Lechner F, Pircher H, Zinkernagel RM (1993) Virus persistence in acutely infected immunocompetent mice by exhaustion of antiviral cytotoxic effector T cells. Nature 362: 758–761. 8469287 10.1038/362758a0

[71] Jackson RJ, Maguire DJ, Hinds LA, Ramshaw IA (1998) Infertility in mice induced by a recombinant ectromelia virus expressing mouse zona pellucida glycoprotein 3. Biol Reprod 58: 152–159. 9472936 10.1095/biolreprod58.1.152

[72] Tscharke DC, Woo WP, Sakala IG, Sidney J, Sette A, et al (2006) Poxvirus CD8+ T-cell determinants and cross-reactivity in BALB/c mice. J Virol 80: 6318–6323. 16775319 10.1128/JVI.00427-06 PMC1488955

[73] Scalzo AA, Farrell HE, Karupiah G (2000) Techniques for studying murine natural killer cells in defense against viral infection. Methods Mol Biol 121: 163–177. 10818725 10.1385/1-59259-044-6:163

