# Supplementary figures and images for: Evidence for Persistence of Ectromelia Virus in Inbred Mice, Recrudescence Following Immunosuppression and Transmission to Naïve Mice
Source: PLoS Pathog. 2015 Dec 23;11(12):e1005342. doi: 10.1371/journal.ppat.1005342 (PMC4689526; doi:10.1371/journal.ppat.1005342)

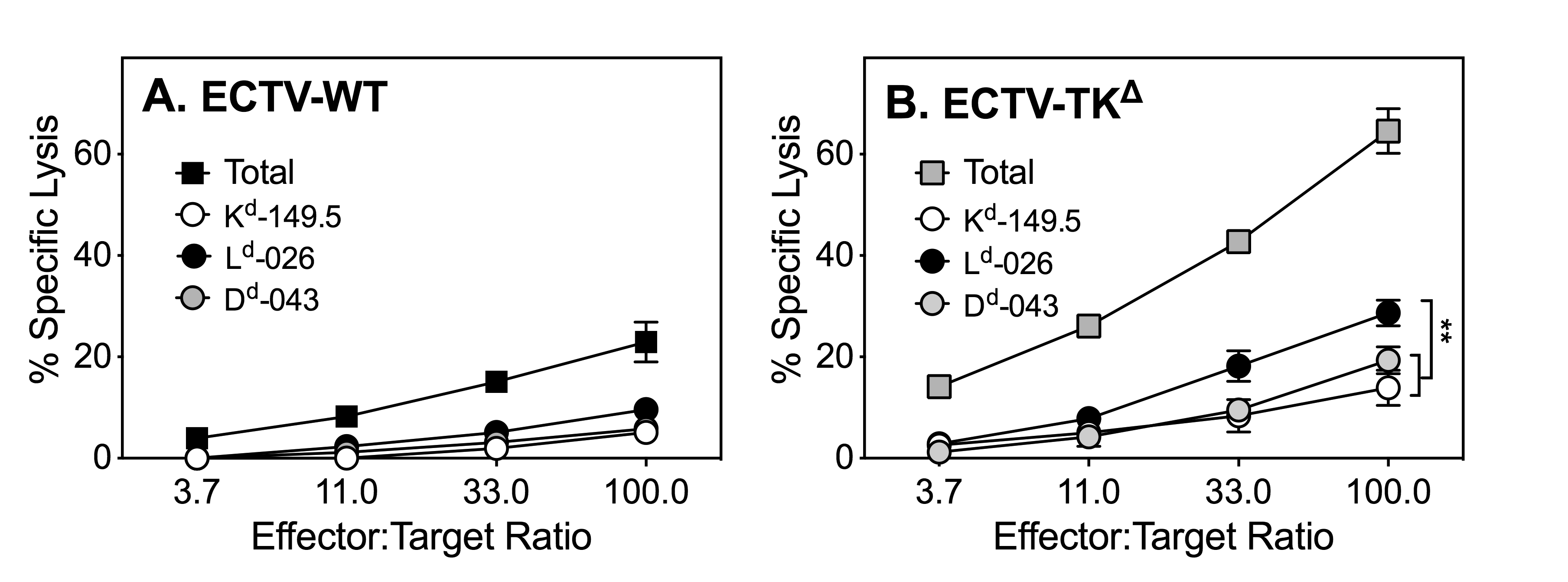

Supplement: S1 Fig — Groups of female BALB/c mice were infected with 500 PFU ECTV-WT or 2 x 10^6^ PFU ECTV-TKΔ s.c., sacrificed on day 7 p.i. and splenic CTL activity was measured. Percent specific lysis of virus-infected (Total) or ECTV CD8 T cell determinant-pulsed, 51Cr-labelled P815 targets by splenocytes from ECTV-WT-infected (A) or ECTV-TKΔ-infected (B) mice. (TIFF) [file ppat.1005342.s001.tiff]

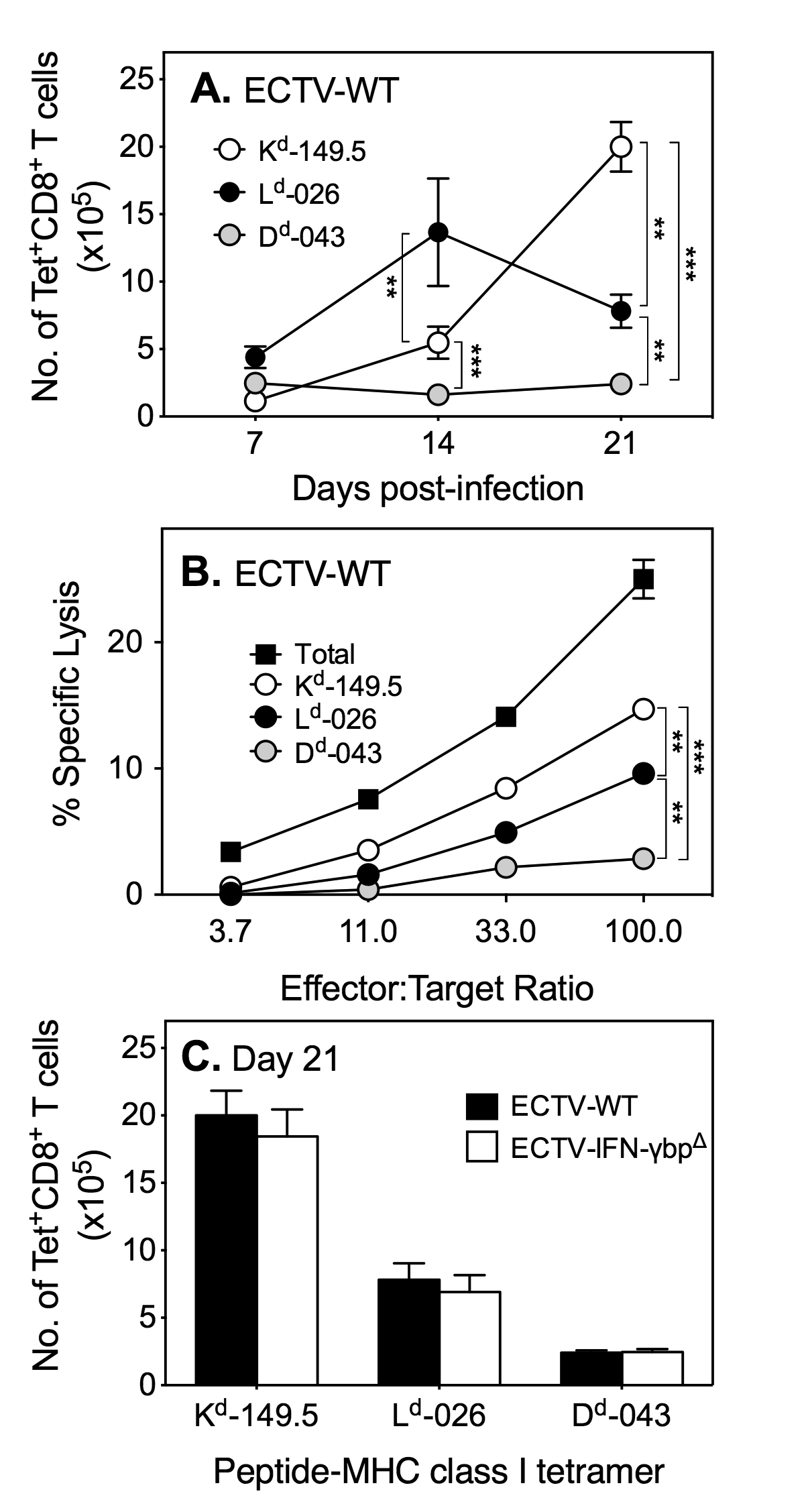

Supplement: S2 Fig — BALB/c mice were infected with 100 PFU of ECTV-WT or 500 PFU of ECTV-IFN-γbpΔ and splenocytes were used to measure CD8 T cell responses at the days indicated. (A) Numbers of peptide-MHC class I tetramer+ CD8 T cells ± SEM at days 7, 14 and 21 p.i. with ECTV-WT. (B) Ex vivo cytolytic activity of splenocytes from ECTV-WT-infected mice 21 days p.i. against ECTV-infected (total) or ECTV peptide determinant-pulsed P815 target cells. P values were obtained by Mann-Whitney U test for the indicated comparisons (A and B): *, P<0.05; **, P<0.01; ***, P<0.001. (C) Numbers of peptide-MHC class I tetramer+ CD8 T cells in BALB/c mice 21 p.i. with 100 PFU ECTV-WT or 500 PFU ECTV-IFN-γbpΔ. (TIFF) [file ppat.1005342.s002.tiff]

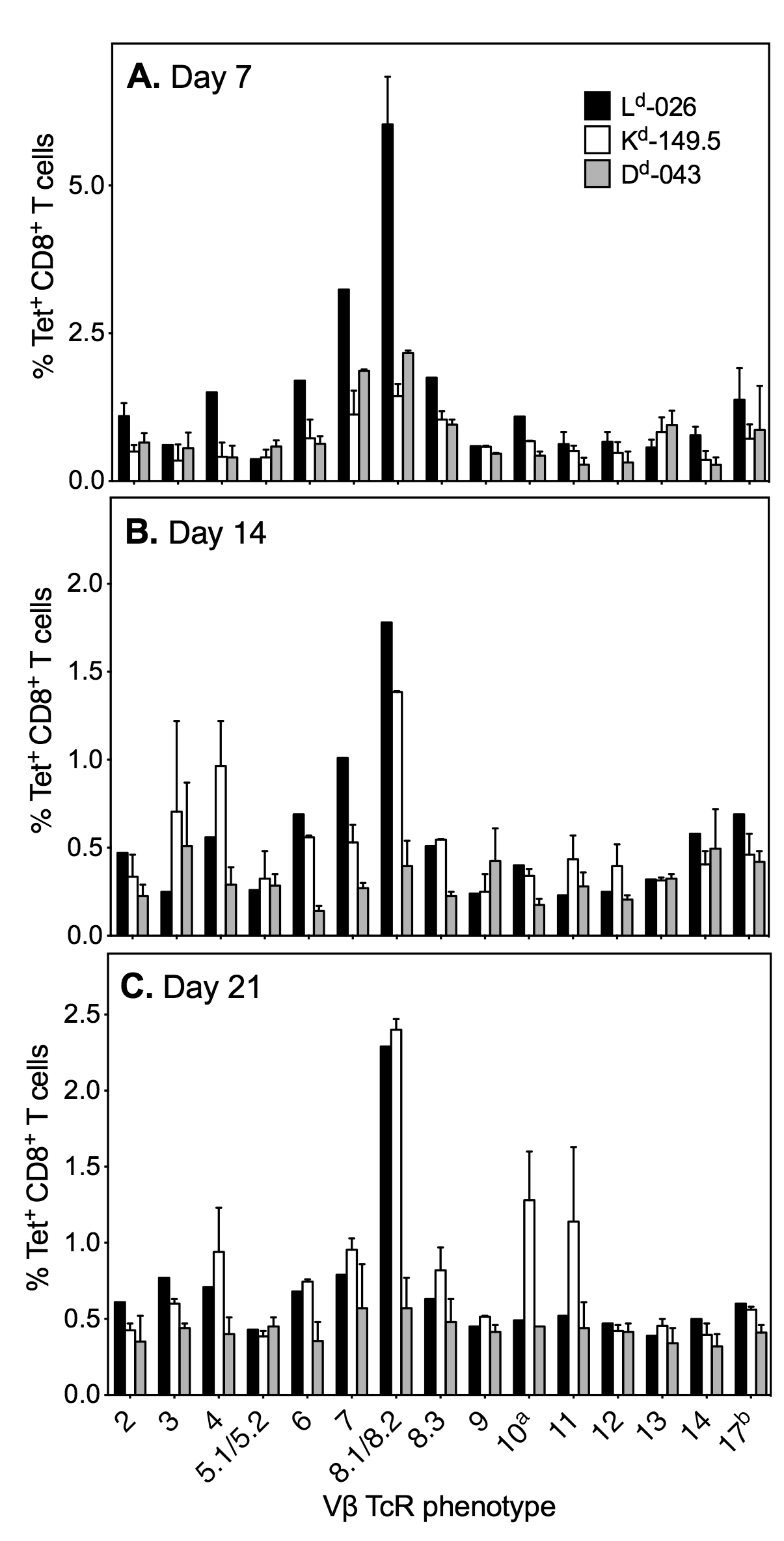

Supplement: S3 Fig — Groups of 6 BALB/c mice were infected with 500 PFU ECTV-IFN-γbpΔ. On the days indicated, mice were sacrificed and splenocytes were co-stained for CD8α (anti-CD8α-APC), mouse VβTCR phenotypes (anti-Vβ TCR-FITC screening panel) and PE-conjugated H-2d tetramers. Events were gated on CD8 T cells and proportions of tetramer-positive CD8 T cells expressing specific Vβ TCR over three weeks p.i. with ECTV-IFN-γbpΔ is shown. Vβ TCR usage at (A) day 7, (B) day 14 and (C) day 21 p.i. Data shown are means ± SEM of two separate animal experiments of 6 mice per group. (TIFF) [file ppat.1005342.s003.tiff]

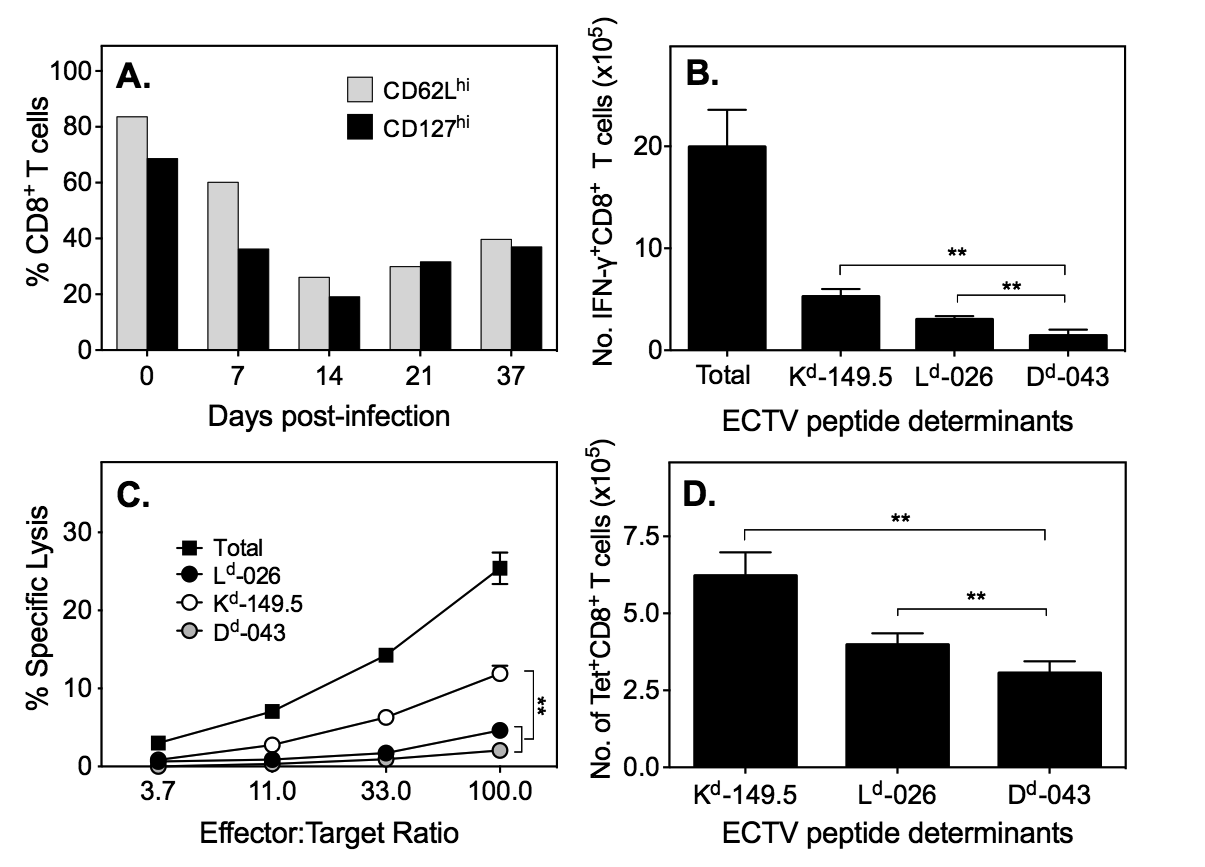

Supplement: S4 Fig — Groups of 5 BALB/c mice were infected with ECTV-IFN-γbpΔ and sacrificed at days 0 (naïve), 7, 14, 21 and 37 p.i.. (A) Relative proportions of CD62Lhi and CD127hi CD8 T cells during early and late primary immune response. (B) Numbers of ECTV-specific (total) and ECTV peptide determinant-specific IFN-γ+ CD8 T cells at day 37 p.i. (C) Ex vivo cytolytic activity of splenocytes from ECTV-IFN-γbpΔ infected mice 37 days p.i. against ECTV-infected (total) or ECTV peptide determinant-pulsed P815 target cells. (D) Numbers of peptide-MHC class I tetramer+ CD8 T cells at day 37 p.i. P values were obtained by Mann-Whitney U test for the indicated comparisons (B, C and D): *, P<0.05; **, P<0.01. (TIFF) [file ppat.1005342.s004.tiff]

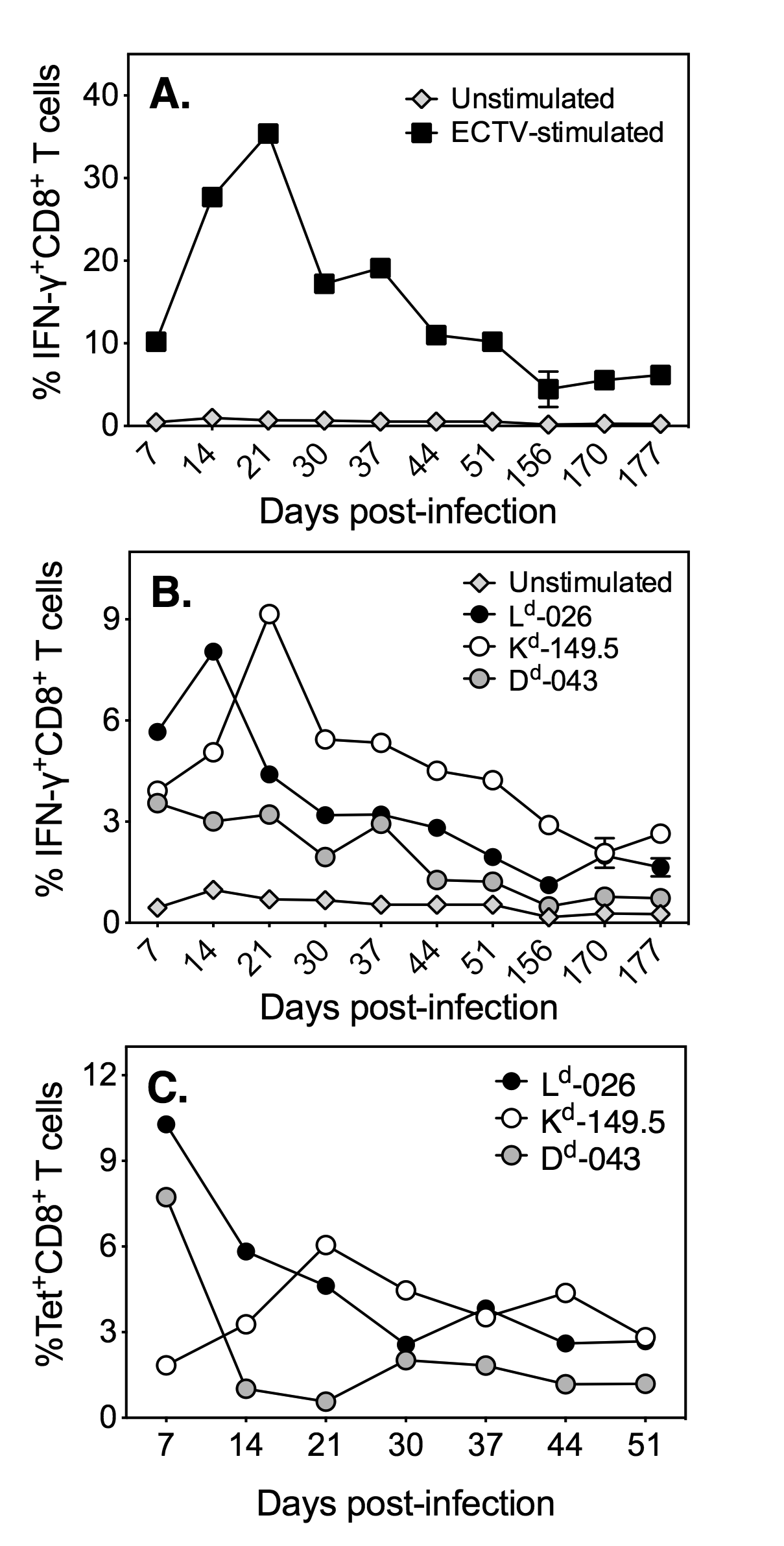

Supplement: S5 Fig — Data on CD8 T cell proportions presented in this figure are derived from the same experiment for which some results are presented in Fig 4. Groups of 6 BALB/c mice were infected with 500 PFU ECTV-IFN-γbpΔ and phenotypic assays undertaken on the days indicated. (A) Kinetics of ECTV-specific (total) IFN-γ+ CD8 T cell proportions, with or without stimulation with ECTV. (B) Kinetics of ECTV peptide determinant-specific IFN-γ+ CD8 T cell proportions. (C) Kinetics of peptide-MHC class I tetramer+ CD8 T cell proportions from day 7 till day 51. (TIFF) [file ppat.1005342.s005.tiff]

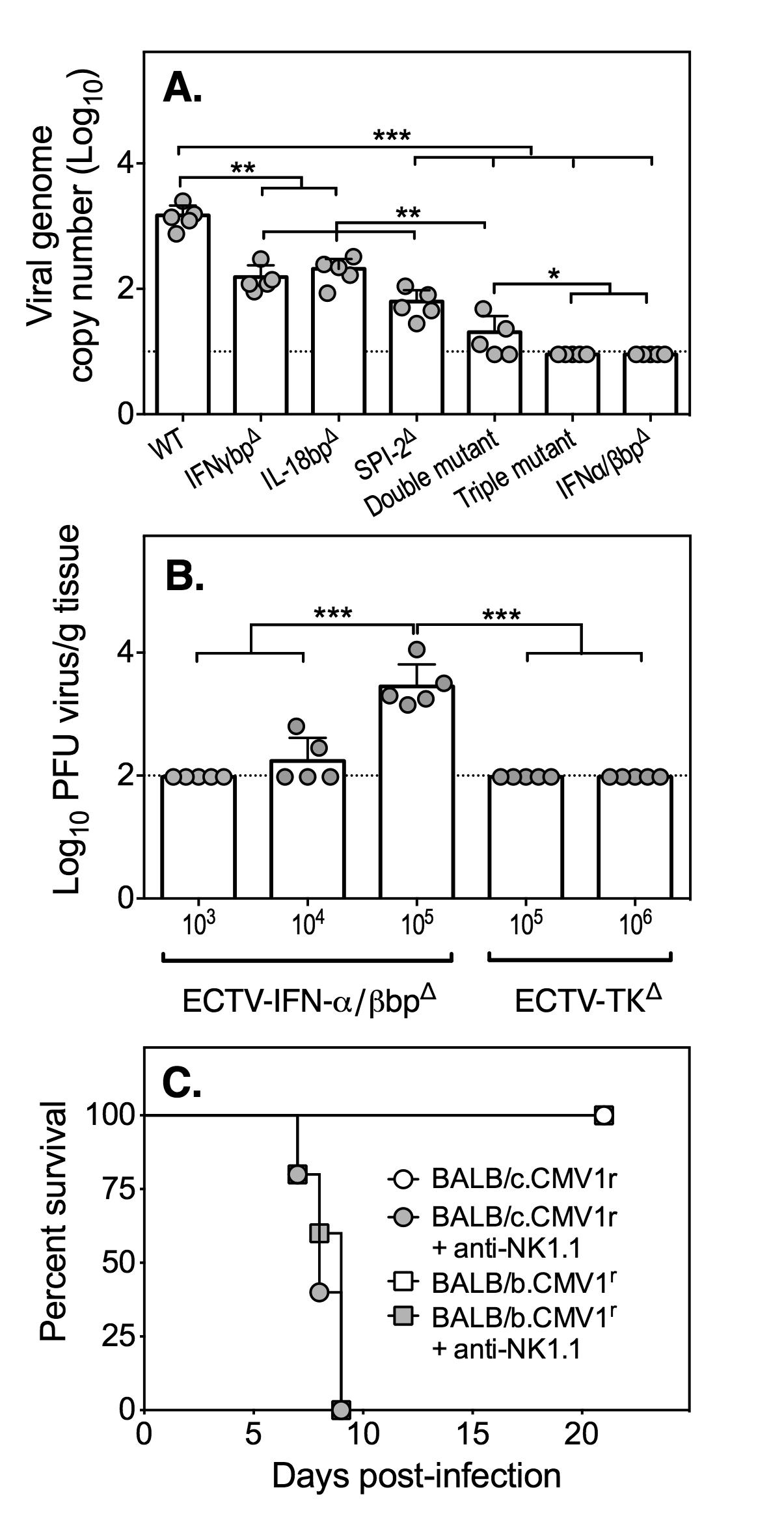

Supplement: S6 Fig — Data presented in this figure is derived from the same experiment for which some results are presented in Fig 5. Groups of 5 female BALB/c mice were infected and sacrificed on day 35 to measure viral load. (A) Virus genome copy numbers in blood of BALB/c mice 35 days p.i. with 100 PFU of WT, single mutant, double mutant or triple mutant ECTV. The limit of virus genome detection is 10 copies and is shown by the dotted line. (B) Viral load in the liver at day 7 p.i. in BALB/c mice infected with varying doses of ECTV-IFN-α/βbpΔ or ECTV-TKΔ. The limit of virus detection is 2 log10 PFU, shown by the dotted line. (C) Depletion of NK cells in the congenic BALB/c.Cmv1r and BALB/b.Cmv1r mice by treatment with anti-NK1.1 mAb overcomes resistance to ECTV-WT infection. The congenic strains but not WT BALB/c or BALB/b mice express the NK1.1 antigen. P values for (A) and (B) were obtained by Mann-Whitney U test: *P<0.05, **P<0.01, and ***P<0.001. P values in (C) were obtained by Log-rank (Mantel-Cox) test: **, P<0.01. (TIFF) [file ppat.1005342.s006.tiff]

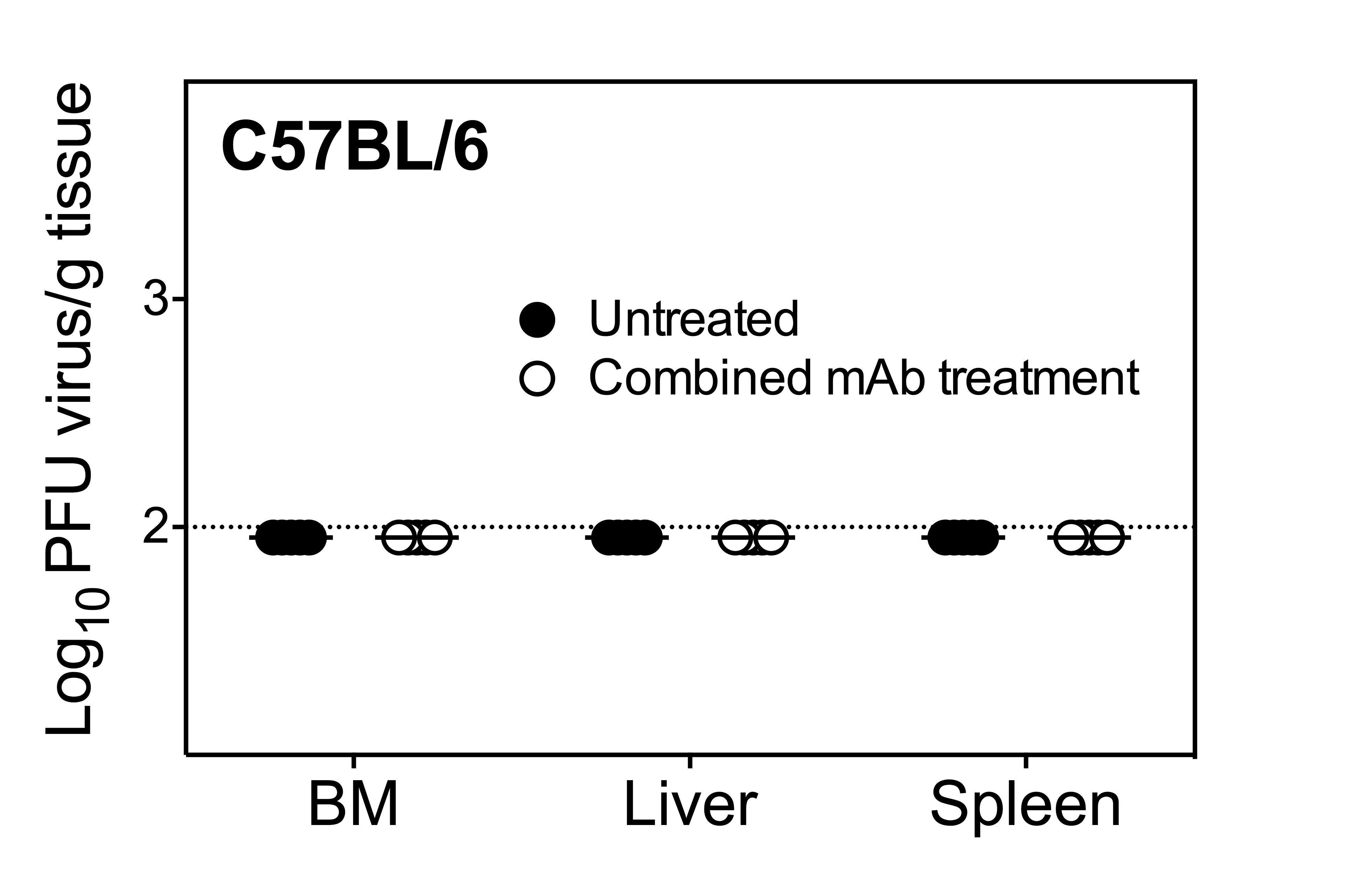

Supplement: S7 Fig — Groups of 5 WT C57BL/6 mice were infected with 1000 PFU of ECTV-WT. Beginning at 80 days p.i, mice were treated with monoclonal antibodies every 2–3 days to deplete NK cells, granulocytes, plasmacytoid dendritic cells, CD4 T cells and CD8 T cells for a period of 3 weeks. Viral load was measured in the indicated organs 3 days after the last treatment (day 104 p.i). (TIFF) [file ppat.1005342.s007.tiff]

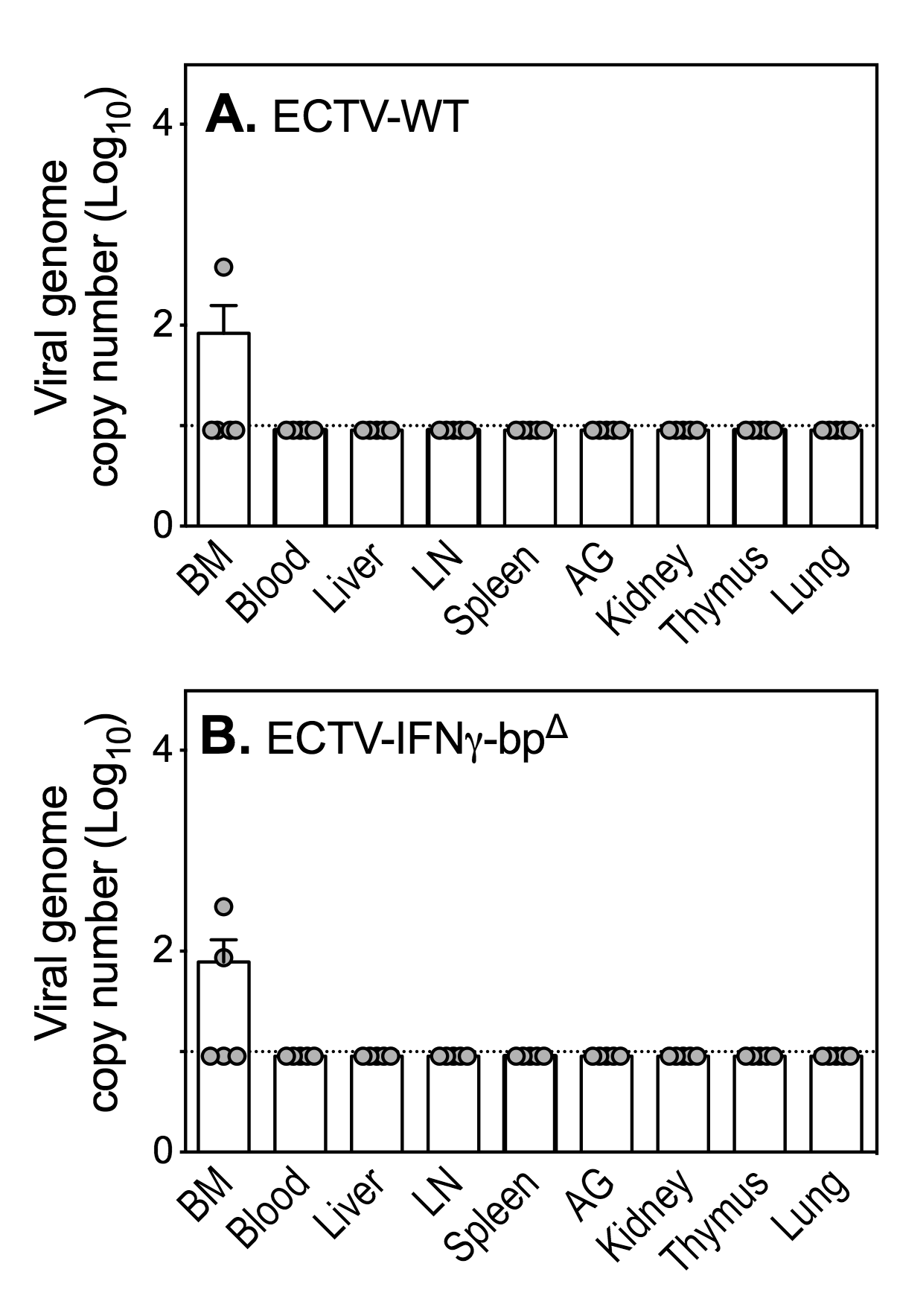

Supplement: S8 Fig — Data presented in this figure is derived from the same experiment for which some results are presented in Fig 6. Groups of 5 WT BALB/c mice were infected with 100 PFU of ECTV-WT or ECTV-IFN-γbpΔ and sacrificed at day 80 p.i. to quantify virus genomes in various organs. Virus genome copy numbers in organs of BALB/c mice infected with (A) ECTV-WT or (B) ECTV-IFN-γbpΔ. (TIFF) [file ppat.1005342.s008.tiff]

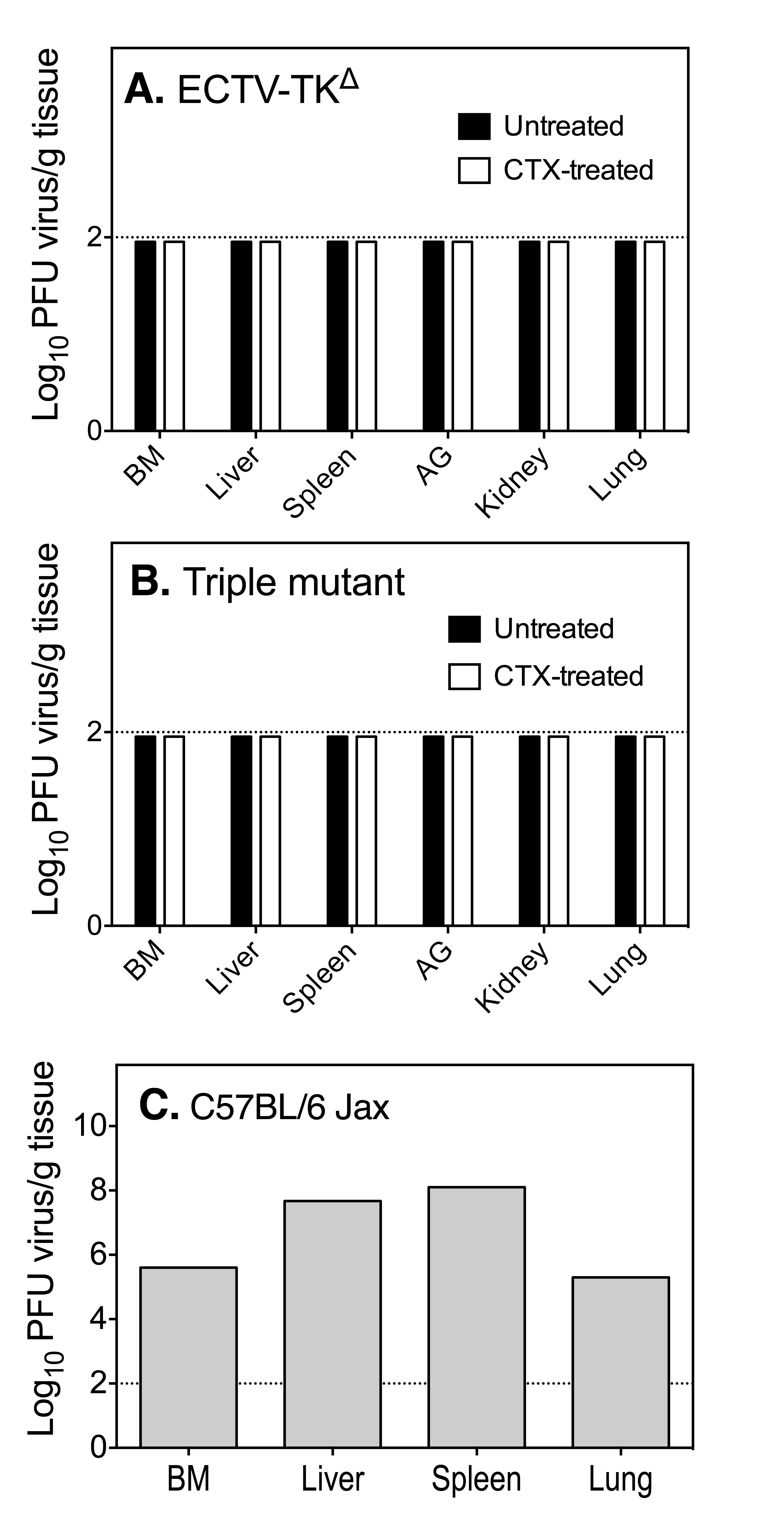

Supplement: S9 Fig — For A and B, groups of 5 WT BALB/c mice were infected with 105 PFU of ECTV-TKΔ or the triple mutant ECTV-IFN-γbpΔ-IL-18bpΔ-SPI-2Δ and subjected to immunosuppression with CTX over 4 weeks. Shown are titers of (A) ECTV-TKΔ and (B) ECTV-IFN-γbpΔ-IL-18bpΔ-SPI-2Δ in the various organs. For C, organs from the one C57BL/6J mice infected for over 80 days with 103 PFU ECTV-WT and treated with CTX that died were collected for determination of viral load. Shown are virus titers in the BM, liver, spleen and lung (C). (TIFF) [file ppat.1005342.s009.tiff]

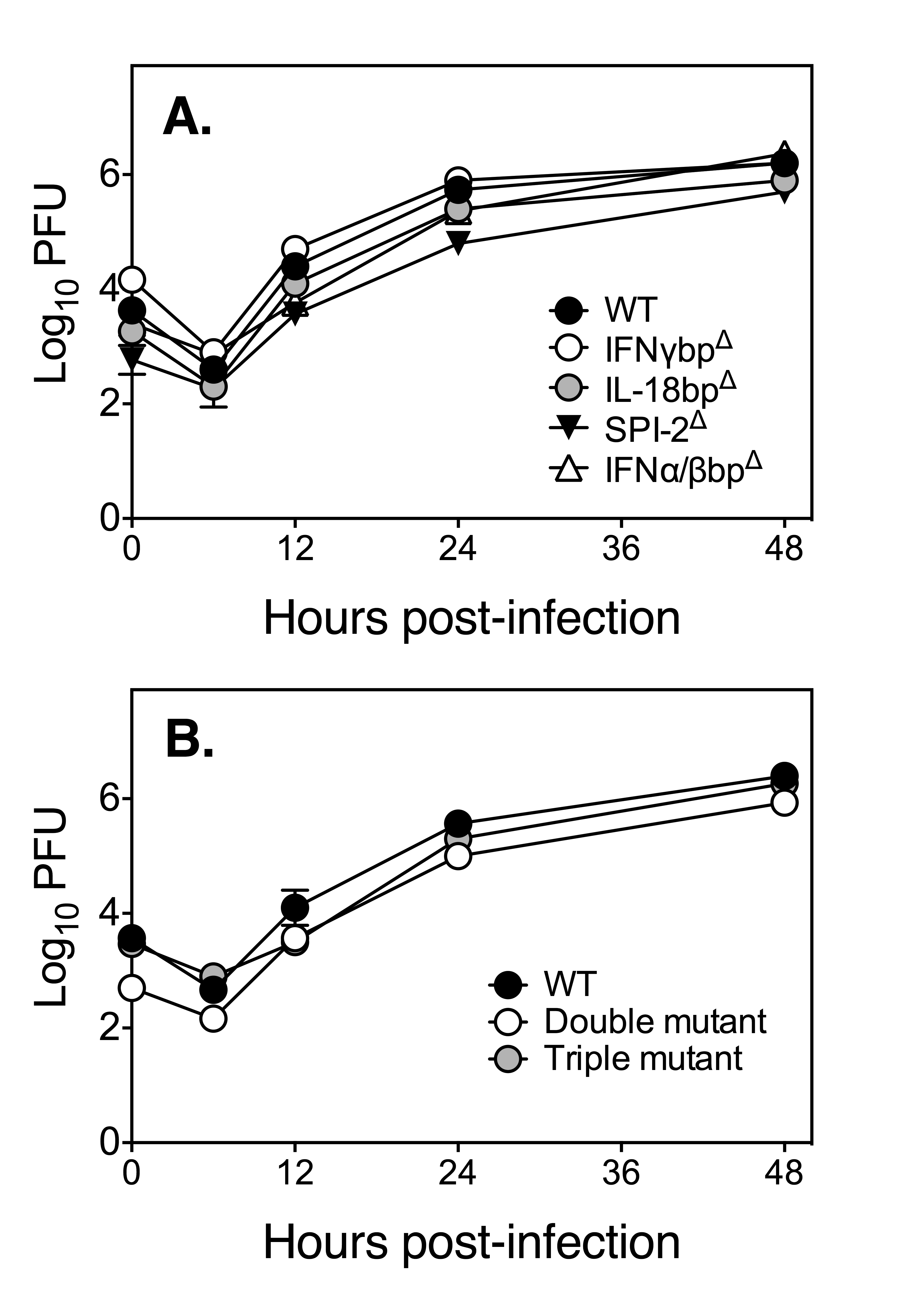

Supplement: S10 Fig — Monolayers of BS-C-1 cells were infected with WT or single mutant viruses (A) or WT, double or triple mutant viruses (B) at 0.1 PFU/ cell in 12-well plates. Cells and supernatant were harvested at the indicated times and the viral load measured by virus plaque assay. Data shown are means ± SD of triplicate cultures. (TIFF) [file ppat.1005342.s010.tiff]
